# A unified allosteric/torpedo mechanism for transcriptional termination on human protein-coding genes

**DOI:** 10.1101/gad.332833.119

**Published:** 2020-01-01

**Authors:** Joshua D. Eaton, Laura Francis, Lee Davidson, Steven West

**Affiliations:** The Living Systems Institute, University of Exeter, Exeter EX4 4QD, United Kingdom

**Keywords:** XRN2, transcriptional termination, CPSF73, polyadenylation signal, RNA polymerase II, PP1, antisense oligo

## Abstract

The allosteric and torpedo models have been used for 30 yr to explain how transcription terminates on protein-coding genes. The former invokes termination via conformational changes in the transcription complex and the latter proposes that degradation of the downstream product of poly(A) signal (PAS) processing is important. Here, we describe a single mechanism incorporating features of both models. We show that termination is completely abolished by rapid elimination of CPSF73, which causes very extensive transcriptional readthrough genome-wide. This is because CPSF73 functions upstream of modifications to the elongation complex and provides an entry site for the XRN2 torpedo. Rapid depletion of XRN2 enriches these events that we show are underpinned by protein phosphatase 1 (PP1) activity, the inhibition of which extends readthrough in the absence of XRN2. Our results suggest a combined allosteric/torpedo mechanism, in which PP1-dependent slowing down of polymerases over termination regions facilitates their pursuit/capture by XRN2 following PAS processing.

## Abstract (precis)

In this study, Eaton et al. examine the validity of the allosteric and torpedo models of transcription termination on protein-coding genes. Using several genomic and molecular assays, the authors propose a model that combines both allosteric/torpedo mechanisms, in which PP1-dependent slowing down of polymerases over termination regions facilitates their pursuit/capture by XRN2 following poly(A) signal processing.

Termination by RNA polymerase II (Pol II) completes the transcription cycle. It serves to recycle Pol II for new rounds of initiation and prevents interference with the transcription of neighboring genes. On protein-coding genes, transcriptional termination is mechanistically connected with 3′ end formation, which involves cleavage and polyadenylation (CPA) (Proudfoot 2011). For CPA, a multiprotein complex is recruited to the polyadenylation signal (PAS), which consists of a hexamer (usually AAUAAA) and a downstream GU (or U)-rich sequence. The 73-kDa component of cleavage and polyadenylation specificity factor (CPSF73) is the endonuclease that cleaves between these two sequences (Mandel et al. 2006). A poly(A) tail is added to the stable upstream cleavage product and the downstream RNA is rapidly degraded. Mutation of the PAS hexamer prevents transcriptional termination—a result that led to two models to explain termination that have been debated ever since (Connelly and Manley 1988; Proudfoot 1989).

The allosteric (or antiterminator) model proposes that transcription of the PAS induces a change in the elongation complex that renders it prone to termination (Logan et al. 1987). This might involve the exchange of associated factors or modification of proteins including Pol II as shown by chromatin immunoprecipitation (ChIP) experiments (Ahn et al. 2004; Kim et al. 2004a). There are also examples of antitermination factors in budding yeast (PC4) and humans (SCAF4 and SCAF8) (Calvo and Manley 2001; Gregersen et al. 2019). Conversely, the 3′ end processing factor, PCF11, can release Pol II from DNA in vitro (Zhang and Gilmour 2006). Allostery may constitute protein modification(s) with phosphorylation-based switches underpinning the 3′ end transition in budding and fission yeast (Schreieck et al. 2014; Kecman et al. 2018; Parua et al. 2018). Specifically dephosphorylation of the Pol II C-terminal domain (CTD) and the SPT5 elongation factor occur after the PAS during an elongation to termination transition. Finally, Pol II conformational changes can result from PAS transcription in purified systems (Zhang et al. 2015).

The torpedo model proposes that the Pol II-associated product of PAS cleavage is degraded by a 5′ → 3′ exonuclease leading to termination (Connelly and Manley 1988; Proudfoot 1989). Evidence for this mechanism was first provided in budding yeast and human cells where mutation or depletion of their respective nuclear 5′ → 3′ exonucleases, Rat1 and XRN2, inhibits termination (Kim et al. 2004b; West et al. 2004). Subsequent studies confirmed the generality of these findings in both organisms (Fong et al. 2015; Baejen et al. 2017; Eaton et al. 2018). An important distinction between the torpedo and allosteric mechanisms is that, in principle, only the former requires PAS cleavage. However, an inactivating point mutation in CPSF73 does not support termination arguing that PAS cleavage cannot readily be bypassed in cells (Eaton et al. 2018).

Given the substantial evidence for both models, it is likely that termination actually employs aspects of each. One possibility is that some genes use an allosteric process with others using XRN2. This might be the case in *C. elegans*, where XRN2 depletion causes termination defects on only a subset of genes (Miki et al. 2017). Another scenario is that allosteric and torpedo processes contribute to a common mode of termination as has been proposed in budding yeast where PCF11 and Rat1 are each required for the others recruitment to genes (Luo et al. 2006). Although human PCF11 is recruited to genes irrespective of XRN2 (Eaton et al. 2018), some PAS-dependent processes could aid XRN2 function. For instance, Pol II pausing downstream from a PAS could facilitate its pursuit by XRN2 especially since pausing enhances termination on reporter plasmids (Gromak et al. 2006).

We investigated the transcriptional termination mechanism in human cells using modified cell lines that allow more rapid depletion of CPSF73 or XRN2 than more commonly used systems. CPSF73 depleted in this manner causes profound readthrough suggesting that its function is essential for termination and that there are no significant fail-safe mechanisms in its absence. CPSF73 is required to slow down Pol II beyond the PAS and for phosphorylation of its CTD on threonine 4 (Thr4p). These slowed-down polymerases pile up beyond the PAS, and their presence, is enriched by rapid depletion of XRN2. Strikingly, longer readthrough can be induced in the absence of XRN2 by inhibiting or depleting protein phosphatase 1 (PP1), identifying a mechanistic basis for the pause. We propose that allosteric events facilitate the pursuit of Pol II and its termination by XRN2 constituting a unified allosteric/torpedo mechanism for transcriptional termination on human protein-coding genes.

## Results

### CPSF73-associated functions are critical for termination on protein-coding genes

**Figure 1.**
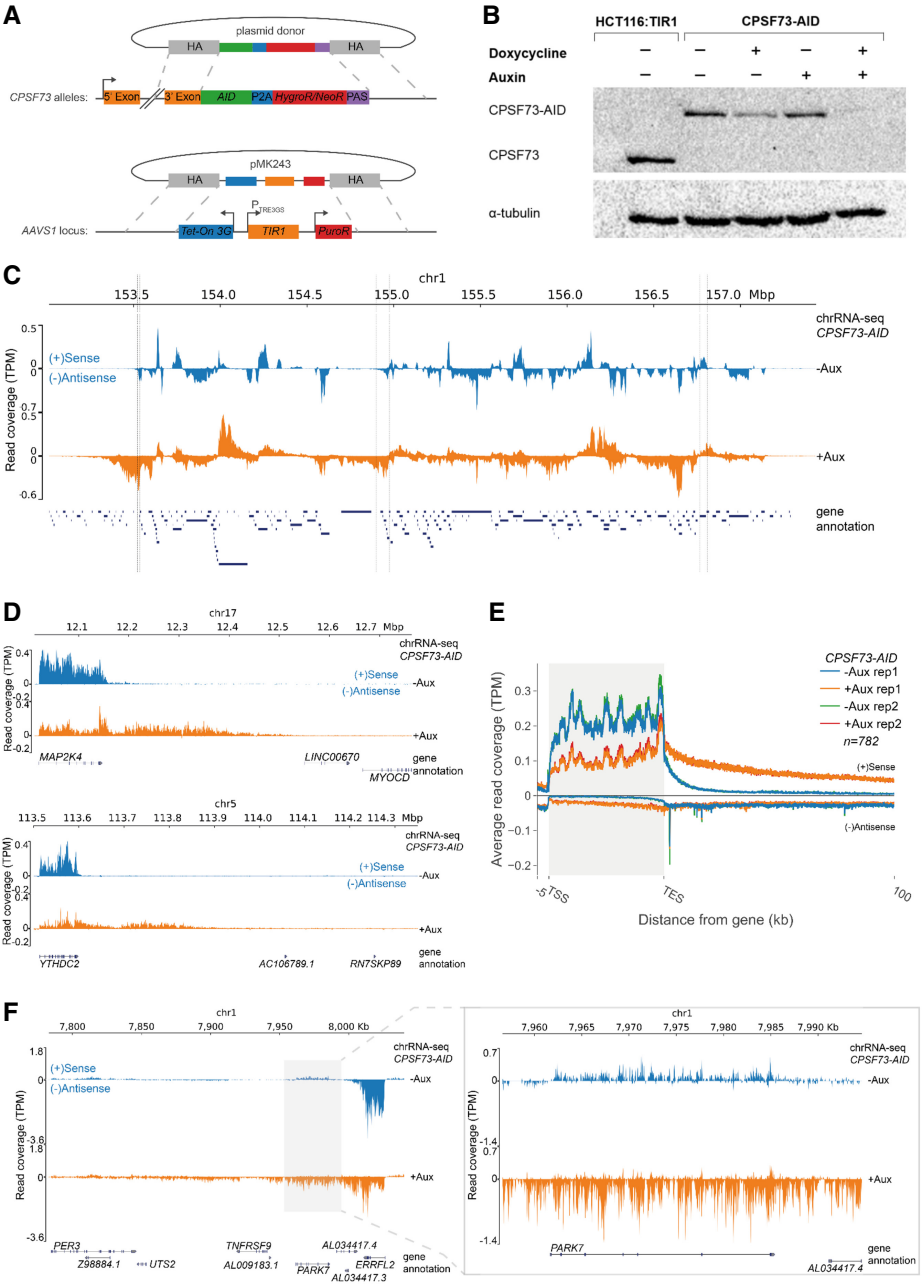
Acute loss of CPSF73 causes profound transcriptional readthrough. (*A*) Diagram depicting the strategy for AID tagging of *CPSF73*. Homology arms (gray) facilitate integration of a cassette containing the AID, separated from Neomycin or Hygromycin resistance genes by a P2A cleavage site and terminated by an SV40 PAS. Dox-inducible *TIR1* is inserted at the *AAVS1* locus. (*B*) Western blot of parental HCT116:TIR1 cells and *CPSF73-AID* cells that were untreated or treated with dox (18 h), auxin (18 h) or dox (18 h) and then auxin (3 h). The *top* panel shows CPSF73 and the *bottom* panel shows the tubulin loading control. (*C*) Chromosomal snapshot from chromatin RNA-seq of *CPSF73-AID* cells treated (orange) or not (blue) with auxin (3 h). Transcription units are seen in control samples (some examples shown with dotted lines). Annotated genes are in blue *below* the snapshot. *Y*-axis scale is transcripts per million (TPM). (*D*) IGV snapshots of *MAP2K4* and *YTHDC2* from the same experiment shown in *C*. (*E*) Metagene plot of expressed genes separated from their neighbors by at least 100 kb in chromatin RNA-seq of *CPSF73-AID* cells treated or not with auxin (3 h). Two biological replicates are plotted. (*F*) Example of transcriptional interference induced in *cis* by CPSF73 loss where readthrough from *ERRFI1* down-regulates the expression of *PARK7*. The *right* panel shows a zoomed in version demonstrating reduced *PARK7* signal coincident readthrough from nearby *ERRFI1*.

We previously found that rapid elimination of XRN2, via an auxin-inducible degron (AID), causes a general termination defect on protein-coding genes (Eaton et al. 2018). In the same study, CPSF73 elimination via an *E. coli*-derived DHFR degron caused more extended readthrough of selected genes, implying a comparatively more important role for its activities in termination. The DHFR degron requires ∼10 h for depletion, whereas XRN2-AID could be depleted faster, so we tagged *CPSF73* with an AID to better-compare CPSF73 and XRN2 functions (Fig.1A; Natsume et al. 2016). AID depletion requires the plant TIR1 protein, which was integrated into HCT116 cells (chosen for their diploid karyotype) to allow its doxycycline (dox)-dependent induction. The Western blot in Figure 1B confirms homozygous tagging of *CPSF73* and that full depletion depends on both dox and auxin. As well as using the same tag, depletion can be achieved in 3 h, providing a more direct comparison to the XRN2-AID system. Note that dox treatment alone induces mild CPSF73 depletion, which may be why we were previously unable to combine CPSF73-AID with constitutive TIR1 expression (Eaton et al. 2018).

To broadly assess the impact of rapid CPSF73 depletion on transcription, we performed RNA-sequencing in mock-treated *CPSF73-AID* cells or the same cells treated with dox and then auxin. Chromatin-associated RNA was sequenced because it is highly enriched in the nascent transcripts that we wished to study. Rapid depletion of CPSF73 caused very obvious and widespread transcriptional readthrough as shown by the chromosome snapshot in Figure 1C. In this 5-Mb view, boundaries of gene transcription are easily observed, but become blurred by profound readthrough following CPSF73 elimination. Zoomed-in tracks of example protein-coding genes (*MAP2K4* and *YTHDC2*) further detail this effect where readthrough is for hundreds of kilobases (Fig. 1D). A metagene plot of transcription across all expressed genes, separated by at least 100 kb, showed that long readthrough is general when CPSF73 is depleted (Fig. 1E). This metagene also shows that CPSF73 loss causes a global reduction in gene body signal, suggesting that its presence positively impacts on transcription.

A termination defect in the absence of CPSF73 is not unexpected; however, the effect here is much greater than previously observed using RNAi (Supplemental Fig. S1A), and more reminiscent of that observed on some genes upon cell stress or viral infection (Vilborg et al. 2015; Heinz et al. 2018). It also reveals a broader range of CPSF73 functions than RNAi depletion, including a role in transcriptional termination of some long noncoding RNAs (Supplemental Fig. S1B,C). The extensive nature of the CPSF73 readthrough also highlights transcriptional interference in *cis*. Figure 1F shows an example whereby readthrough from *ERRFL1* reduces the expression of the convergent *PARK7* gene. Finally, CPSF73 depletion did not affect integrator-dependent snRNA gene termination demonstrating the specificity of these effects (Supplemental Fig. S1D). We conclude that functions of CPSF73 are indispensable for Pol II termination on protein-coding genes.

### Predicted RNA products of XRN2-independent termination are not abundant

**Figure 2.**
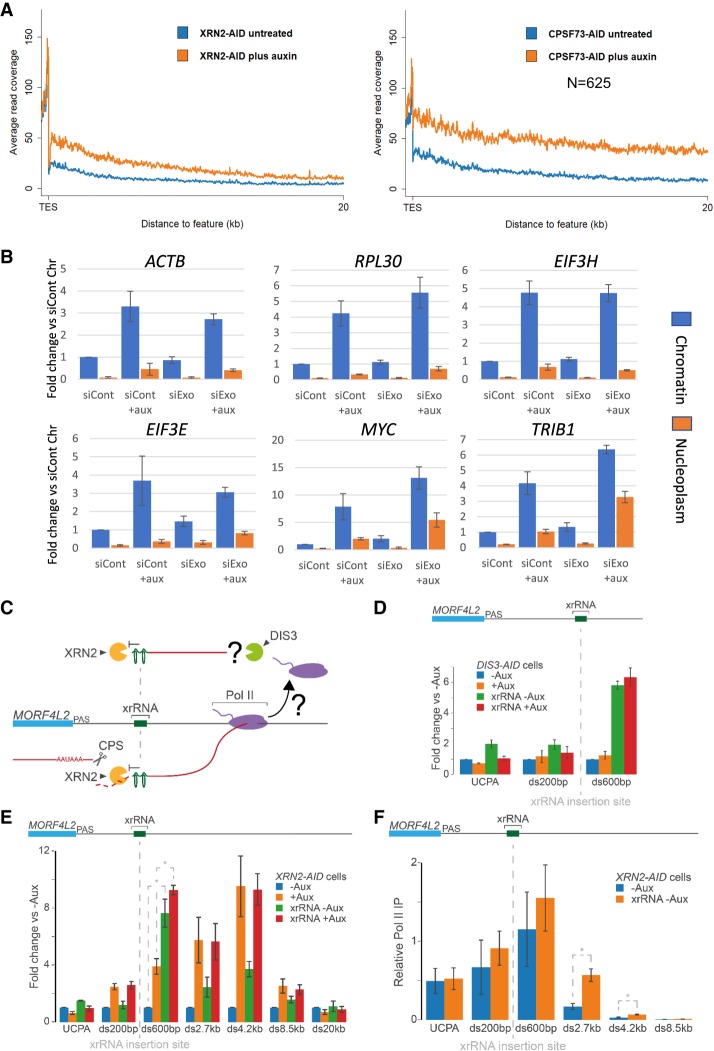
XRN2-independent termination is not readily apparent. (*A*) Metagene plots of expressed genes separated from their neighbors by at least 20 kb from nuclear RNA-seq of *CPSF73-AID* and *XRN2-AID* cells treated or not with auxin. Note that the *n* number is lower here than for the 100-kb window in Figure 1E due to stricter exclusion criteria applied to our previously generated *XRN2-AID* data (see Supplemental Material). (*B*) qRT-PCR analysis of *ACTB*, *MYC*, *RPL30*, *TRIB1*, *EIF3E*, and *EIF3H* 3′ flanking RNA in chromatin-associated and nucleoplasmic RNA from *XRN2-AID* cells transfected with control or exosome siRNAs before treatment or not with auxin (2 h). The graph shows fold change in RNA levels relative to the chromatin fraction of control cells following normalization to spliced ACTB. *n* = 3. Error bars are SEM. (*C*) Schematic illustrating the insertion of xrRNA into the 3′ flank of *MORF4L2* and its predicted effects on transcript degradation. If there is termination in the absence of 5′ → 3′ degradation this should be revealed by acute DIS3 depletion. CPS is cleavage and polyadenylation site. (*D*) qRT-PCR analysis of *MORF4L2* readthrough in unmodified *DIS3-AID* cells and *DIS3-AID* cells modified at *MORF4L2* by xrRNA insertion, then treated or not with auxin (2 h). The graph shows fold change in RNA at each amplicon relative to unmodified *DIS3-AID* cells not treated with auxin after normalizing to spliced ACTB. *n* = 3. Error bars are SEM. UCPA detects transcripts not cleaved at the PAS. (*E*) qRT-PCR analysis of *MORF4L2* readthrough in unmodified *XRN2-AID* cells and *XRN2-AID* cells modified at *MORF4L2* by xrRNA insertion, then treated or not with auxin (2 h). The graph shows fold change in RNA at each amplicon compared with unmodified *XRN2-AID* cells untreated with auxin after normalizing to spliced ACTB. *n* = 3. Error bars are SEM. (*) *P* < 0.05 illustrates the xrRNA effectiveness exemplified at ds600. (*F*) Pol II ChIP analysis on unmodified or xrRNA-modified *MORF4L2* in *XRN2-AID* cells that were not subject to treatment. Graph shows relative Pol II IP after normalizing to the *ACTB* ds1.7 kb amplicon, which is unmodified in both cell lines. *n* = 3. Error bars are SEM. (*) *P* < 0.05.

The very long readthrough seen without CPSF73 contrasts with our previous measurements of Pol II occupancy in the absence of XRN2 (Eaton et al. 2018). This showed a more moderate termination defect, defined as such because readthrough and Pol II signal eventually reduce to background levels even after XRN2 depletion. To compare XRN2 and CPSF73 effects, we analyzed our previously generated nuclear RNA-seq from *XRN2-AID* cells and newly generated nuclear RNA-seq from C*SF73-AID* cell samples treated or not with auxin (Fig. 2A). Metagene plots were generated for all expressed genes separated from their neighbors by at least 20 kb. Loss of XRN2 shows clear stabilization of readthrough RNA just downstream from the PAS as we previously reported (Eaton et al. 2018), but this effect dissipated by 20 kb. In the absence of CPSF73, there was also strong stabilization of readthrough RNA, but this effect was maintained for the full 20 kb. Thus, there is generally longer readthrough seen when CPSF73 is depleted versus when XRN2 is lost. We focused subsequent efforts on establishing the basis for this difference as a means to understand the termination mechanism.

A reasonable explanation for this difference is XRN2-independent termination that still requires CPSF73 (Eaton and West 2018). This mechanism would release the RNA 3′ end from the terminated polymerase, leaving it vulnerable to 3′ → 5′ degradation by the exosome, which degrades many noncoding RNAs shortly after their synthesis (Preker et al. 2008). To test this, we transfected *XRN2-AID* cells with control siRNAs or siRNAs to deplete the exosome (EXOSC3 and EXOSC10) before treatment or not with auxin to remove XRN2. Chromatin-associated and nucleoplasmic RNA was isolated as we anticipated that termination products might be released from chromatin. To check the validity of this approach, quantitative reverse transcription and PCR (qRT-PCR) was performed to detect a PROMPT transcript upstream of *RBM39,* which is a well-characterized exosome substrate. This was strongly stabilized by exosome depletion, validating siRNA function, with the effect largest in the nucleoplasmic fraction (Supplemental Fig. S2A).

3′ flanking RNA derived from six protein-coding genes was then analyzed (Fig. 2B). As expected, all were up-regulated by loss of XRN2; however, depletion of the exosome alone had little effect on their levels or distribution. This argues that their degradation is primarily in the 5′ → 3′ direction. Codepletion of XRN2 and the exosome did not enhance RNA levels beyond those seen by eliminating XRN2 alone in most cases. *MYC* and *TRIB1* were two exceptions that showed an additional nucleoplasmic increase, which may indicate some XRN2-independent termination on those genes. However, these examples were still less sensitive to exosome loss than the *RBM39* PROMPT control. In our view, these data do not provide convincing evidence for efficient termination in the absence of XRN2.

These findings imply that degradation of 3′ flanking RNA is commonly unidirectional. To probe this more sensitively, we used CRISPR–Cas9 to edit the *MORF4L2* gene to include an XRN-resistant RNA (xrRNA). Isolated from West Nile virus, the xrRNA forms a structure that impairs 5′ → 3′ exonucleases and can be used to probe the directionality of RNA decay (Fig. 2C; Chapman et al. 2014; Horvathova et al. 2017). *MORF4L2* was chosen because it is well expressed and, being on the X chromosome, requires only one editing event to fully modify. It behaves typically insofar as its readthrough transcription is longer in the absence of CPSF73 versus XRN2 (Supplemental Fig. S2B). We performed this gene editing in our recently described *DIS3-AID* cells in which the major catalytic subunit of the exosome, DIS3, can be rapidly depleted by auxin (Davidson et al. 2019). Total RNA was isolated from xrRNA-modified or unmodified *DIS3-AID* cells treated or not with auxin and qRT-PCR used to assay the levels of *MORF4L2* RNA upstream of and downstream from the xrRNA insertion (Fig. 2D). Similar levels of RNA were obtained upstream of the xrRNA under all conditions; however RNA downstream from the xrRNA was stabilized consistent with impaired 5′ → 3′ degradation. This stabilization was not exacerbated by DIS3 loss again suggesting unidirectional decay and infrequent XRN2-independent transcriptional termination. Importantly, DIS3 elimination strongly stabilized other exosome substrates in the same samples (Supplemental Fig. S2C).

### An XRN-resistant RNA does not induce profound readthrough

Another explanation for why readthrough is short in the absence of XRN2 could be the presence of alternative 5′ → 3′ exonucleases or trace levels of XRN2 remaining after auxin treatment. To test this, we used CRISPR–Cas9 to insert the XRN-resistant RNA (xrRNA) downstream from *MORF4L2* in *XRN2-AID* cells to inhibit 5′ → 3′ degradation generally. RNA was isolated from *XRN2-AID* cells modified or unmodified at *MORF4L2* following treatment or not with auxin. qRT-PCR analysis of readthrough transcription showed that RNA between the PAS and xrRNA was stabilized only when XRN2 was depleted as expected (Fig. 2E). Similar to Figure 2D, RNA downstream from the xrRNA was stabilized even in the presence of XRN2 demonstrating that the xrRNA inhibits 5′ → 3′ degradation. Depletion of XRN2 from these cells produced the expected readthrough but its extent (positions beyond ds600) was not significantly greater than in unmodified cells depleted of XRN2. It therefore seems unlikely that alternative 5′ → 3′ exonucleases generally participate in termination.

### Efficient pursuit of Pol II by XRN2 is important for termination

The observation that XRN2 initiates degradation at the PAS but is impeded beyond the xrRNA allows a key prediction of the torpedo model to be tested: that Pol II capture, rather than simply degrading RNA, is critical. Specifically, because the xrRNA impedes degradation that has already been initiated at the PAS, it should only result in a termination defect if the continued pursuit of Pol II is important. We performed Pol II ChIP on *MORF4L2* in xrRNA-modified and unmodified *XRN2-AID* cells without depletion of XRN2 (Fig. 2F). The presence of the xrRNA resulted in significantly enhanced Pol II occupancy downstream from its position (2.7 kb and 4.2 kb), strongly suggesting that XRN2 must catch Pol II (or at least get close to it) to terminate it.

### Polymerases accumulate over termination regions in the absence of XRN2

**Figure 3.**
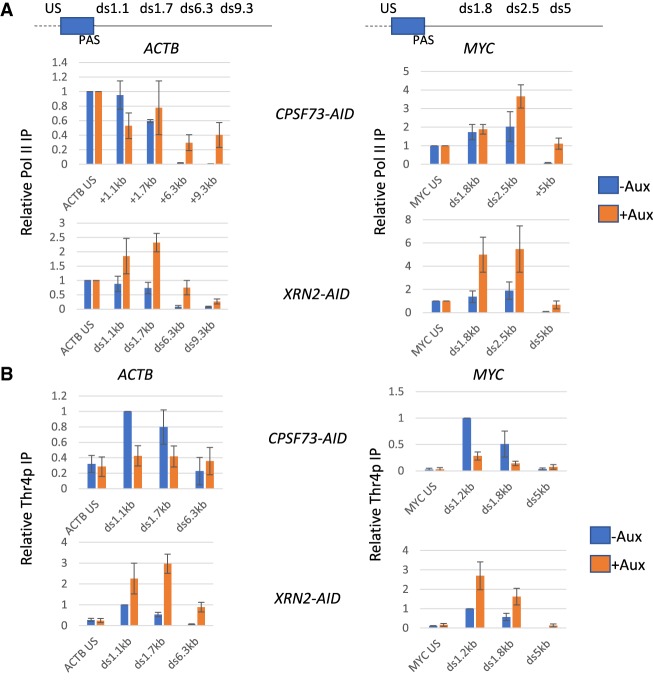
Pol II piles up beyond the PAS when XRN2 is depleted. (*A*) Pol II ChIP analysis on *ACTB* and *MYC* performed in *CPSF73-AID* or *XRN2-AID* cells treated or not with auxin (3 h). Graph shows relative Pol II IP normalized to ACTB US/MYC US in each sample. *n* = 3. Error bars are SEM. (*B*) Thr4p ChIP analysis on *ACTB* and *MYC* performed in *CPSF73-AID* or *XRN2-AID* cells treated or not with auxin (3 h). Graph shows relative Thr4p IP normalized ACTB ds1.1 kb and MYC ds1.2 kb in the respective cell lines not treated with auxin. *n* = 3. Error bars are SEM.

Data so far show that CPSF73 loss causes runaway readthrough, which is much less extensive when XRN2 is depleted. This difference is not due to efficient XRN2-independent mechanisms or other 5′ → 3′ exonucleases, active when XRN2 is eliminated. We therefore hypothesized that Pol II accumulating beyond the PAS when XRN2 is eliminated is slowed rather than terminated and that this is why readthrough is shorter than in the absence of CPSF73. To interrogate this, we performed Pol II ChIP on *XRN2-AID* and *CPSF73-AID* cells treated or not with auxin using *MYC* and *ACTB* as model genes (Fig. 3A). Auxin was added for 3 h in both cases to eliminate/minimize any effects of different depletion times. Loss of CPSF73 or XRN2 induces a termination defect on both genes as expected. The effect of CPSF73 loss was greatest at positions furthest downstream from the PAS, whereas the XRN2 effect was more prominent at positions within 2.5 kb of the PAS. This difference is consistent with the idea that polymerases pile up over termination regions in the absence of XRN2 but not when CPSF73 is depleted. This pile up is likely due to slow elongation rather than complete arrest because 3′ flanking region Pol II can still incorporate 4-thiouridine in the absence of XRN2 (Supplemental Fig. S3A).

We next assayed whether the difference in Pol II behavior caused by XRN2 vs CPSF73 depletion is associated with any changes in its modification status. The CTD of the largest Pol II subunit is heavily modified during the transcription cycle (Eick and Geyer 2013). Thr4p is the most obvious candidate for CPSF73-dependent modification as it predominantly occurs after the PAS and its position is shifted downstream when CPSF73 is depleted by RNAi (Schlackow et al. 2017). We assayed Thr4p on *ACTB* and *MYC* in *CPSF73-AID* and *XRN2-AID* cells grown with or without auxin and observed its expected enrichment beyond the PAS in control samples (Fig. 3B). XRN2 loss caused an increase in Thr4p signal downstream from the PAS for both genes similar to the increase in total Pol II occupancy observed in Figure 3A; however, Thr4p is reduced when CPSF73 is eliminated. Thr4p is therefore downstream from CPSF73 activity but upstream of XRN2-dependent transcriptional termination, revealing a chemical difference between Pol II occupying 3′ flanking regions upon CPSF73 versus XRN2 depletion.

### Polymerases accumulate over termination regions in the absence of XRN2 but transcribe through them when CPSF73 is eliminated

Reduced Pol II occupancy beyond the PAS is usually interpreted as consequential of transcriptional termination. Our data argue that XRN2 depletion enriches polymerases that can neither elongate well nor terminate. We designed an experiment to test this more directly using a hepatitis δ ribozyme that cleaves very efficiently and can be inactivated by a single mutation (δRZ[WT/MT]) (Fong et al. 2009). RZ cleavage produces an upstream product that is degraded from its 3′ end and a downstream product that cannot be degraded due to a 5′OH (Stevens and Maupin 1987; Muniz et al. 2015). Transcription of the RZ will result in a stable downstream product, the presence of which can be used to report instances of transcription beyond its position. *RBM3* was modified with (δRZ[WT/MT]) in *CPSF73-AID* and *XRN2-AID* cells and was chosen as an alternative to *MORF4L2* (it is also X-chromosomal). The RZ was inserted just downstream from where termination normally occurs and loss of XRN2 induces some transcription beyond the insertion site (Supplemental Fig. S3B). If, in the absence of XRN2, the ultimate position of Pol II occupancy defines an auxiliary termination site, then most Pol II should transcribe the RZ when XRN2 is depleted. If instead most Pol II accumulates upstream of the RZ when XRN2 is eliminated only a fraction will transcribe beyond it. *CPSF73-AID* cells act as a control because its depletion induces profound readthrough beyond the RZ insertion site (Supplemental Fig. S3B).

**Figure 4.**
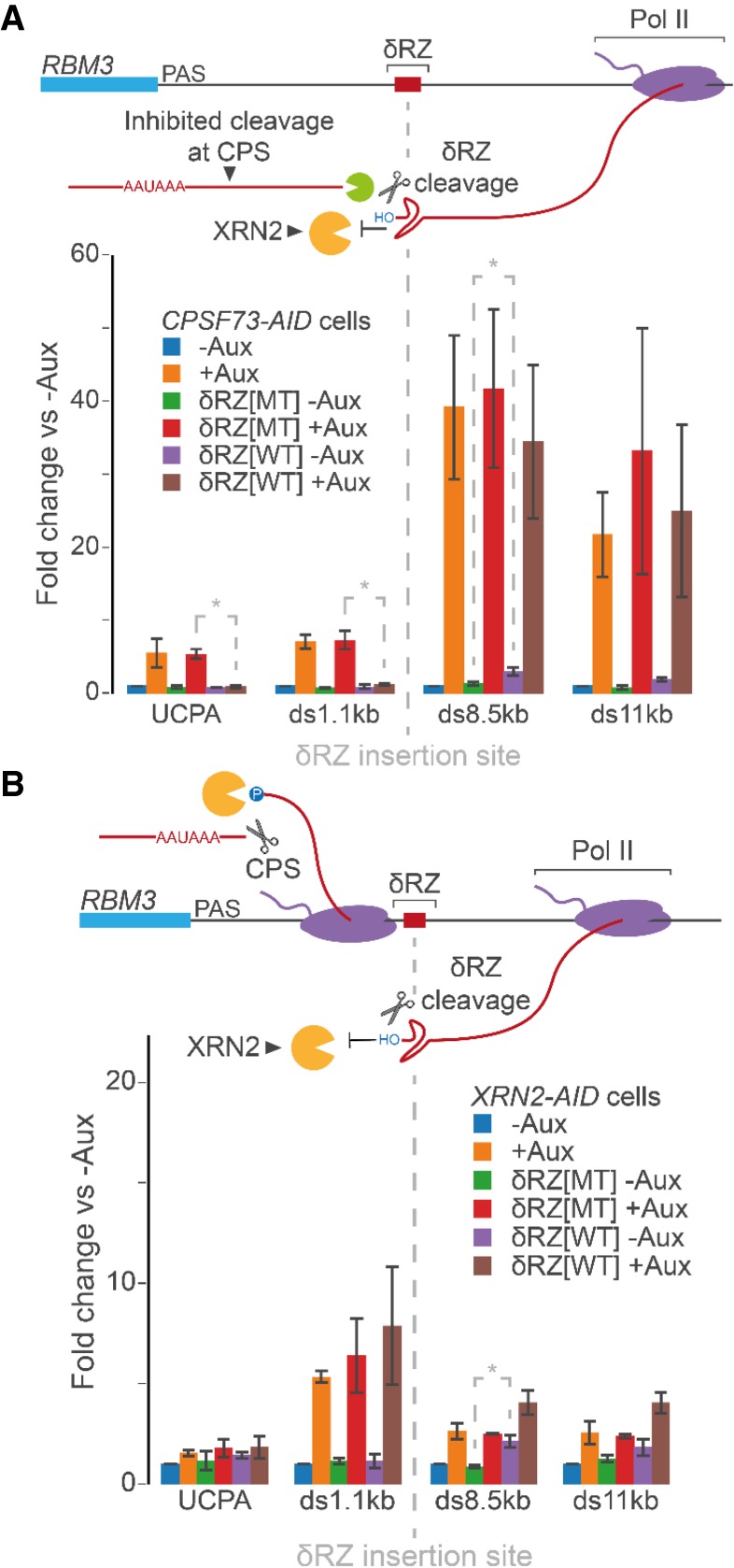
Pol II piles up over termination regions when XRN2 is depleted whereas CPSF73 loss induces runaway transcription. (*A*) qRT-PCR analysis of *RBM3* readthrough in unmodified *CPSF73-AID* cells and *CPSF73-AID* cells modified at *RBM3* by addition of δRZ[WT/MT] and then treated or not with auxin (3 h). The schematic shows the RZ insertion and predicted impact on RNA degradation. The graph shows fold change in RNA over each amplicon relative to unmodified *CPSF73-AID* cells not treated with auxin after normalising to spliced ACTB. *n* ≥ 3. Error bars are SEM. (*) *P* < 0.05 to highlight significance of smaller fold effect changes commented on in the main text. UCPA detects transcripts uncleaved at the PAS. (*B*) qRT-PCR analysis of *RBM3* readthrough in unmodified *XRN2-AID* cells and *XRN2-AID* cells modified at *RBM3* by addition of δRZ[WT/MT] and then treated or not with auxin (3 h). The graph shows fold change in RNA at each amplicon relative to unmodified *XRN2-AID* cells after normalizing to spliced ACTB. *n* ≥ 3. Error bars are SEM. (*) *P* < 0.05 to highlight significance of smaller fold effect changes commented on in the main text.

qRT-PCR was performed to look at readthrough in δRZ[WT/MT]-modified *CPSF73-AID* cells and unmodified *CPSF73-AID* cells treated or not with auxin (Fig. 4A). In unmodified cells, CPSF73 loss induced the expected increase in transcriptional readthrough causing a 25-fold to 50-fold increase in signal at 8.5 and 11 kb beyond the *RBM3* PAS. The result was similar when *RBM3* was modified with δRZ[MT]. With δRZ[WT], there was again strong readthrough when CPSF73 was lost. Importantly, the RZ cleaves in this setting because its upstream product does not accumulate when CPSF73 is depleted from δRZ[WT] samples but shows robust up-regulation in the presence of δRZ[MT] (see UCPA and 1.1-kb amplicons). As expected, the 3′ cleavage product cannot be degraded as its accumulation is similar in δRZ[MT] and δRZ[WT] scenarios.

In *XRN2-AID* cells, δRZ[MT]-modification of *RMB3* gives a similar result to unmodified *XRN2-AID* cells in that XRN2 depletion leads to stabilization of 3′ flanking region RNA (Fig. 4B). For the δRZ[WT], there is mild up-regulation of the downstream 8.5-kb amplicon even when XRN2 is present. This is likely due to a small fraction of Pol II transcribing beyond its position as a similarly modest stabilization is also seen here in untreated *CPSF73-AID* cells modified by δRZ[WT]. Compared with loss of CPSF73, XRN2 depletion has much less impact on readthrough beyond the δRZ[WT]. This supports the idea derived from xrRNA-modified *MORF4L2* that alternate means of 5′ → 3′ degradation do not promote efficient termination when XRN2 is depleted. Finally, XRN2 depletion also results in stabilization of RNA upstream of the RZ instead of the destabilization seen without CPSF73, suggesting that comparatively few polymerases go beyond the RZ in its absence. Thus, the frontier of Pol II signal seen when XRN2 is lost is unlikely to represent an auxiliary termination site and is more likely to reflect the forefront of polymerases accumulated upstream of it. Such accumulation of Pol II beyond the PAS upon XRN2 depletion is general, based on analysis of our previously published mNET-seq in cells lacking XRN2 (Supplemental Fig. S3C).

### PP1 activity aids termination by XRN2

**Figure 5.**
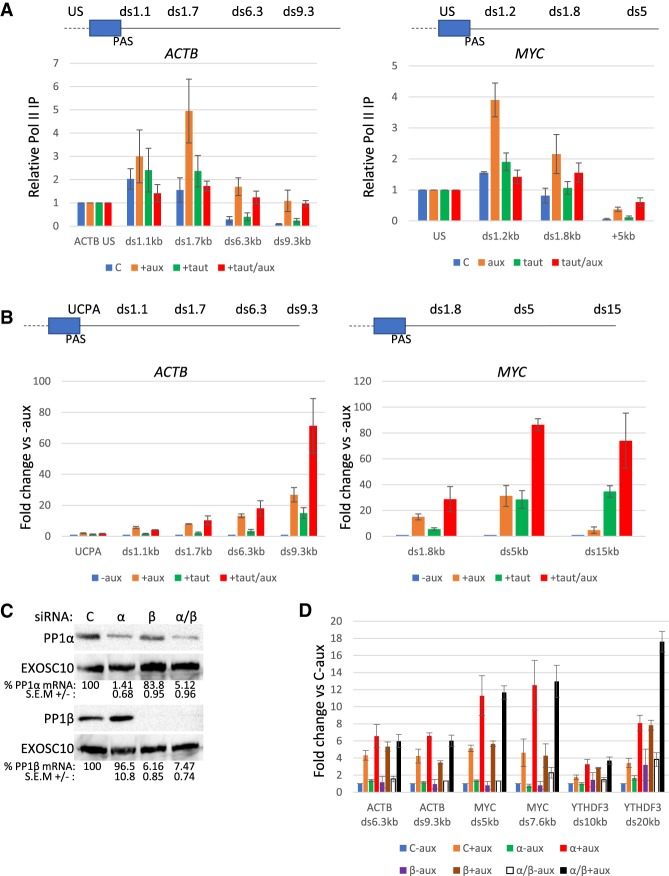
PP1 activity underpins the piling up of Pol II in the absence of XRN2. (*A*) Pol II ChIP on *ACTB* and *MYC* performed in *XRN2-AID* cells that were either untreated or treated with auxin, tautomycetin or both (auxin 2 h, tautomycetin 18 h). Graph shows Pol II IP relative to ACTB US/MYC US for each sample. *n* = 3. Error bars are SEM. (*B*) qRT-PCR analysis of *ACTB* and *MYC* readthrough transcription in *XRN2-AID* cells that were either untreated or treated with auxin, tautomycetin, or both (auxin 2 h, tautomycetin 18 h). Graph shows RNA fold change at each amplicon relative to untreated (-aux) cells following normalization to spliced ACTB. *n* = 3. Error bars are SEM. (*C*) Western blotting of *XRN2-AID* cells treated with control, PP1α, PP1β, or PP1α and β siRNAs. EXOSC10 is shown as a loading control. mRNA levels (*n* = 3) are shown under each blot relative to those in control siRNA treated cells (shown as 100%) after normalization to spliced ACTB transcripts. (*D*) qRT-PCR analysis of *ACTB, MYC*, and *YTHDF3* readthrough transcription in *XRN2-AID* cells that were treated with control, PP1α, PP1β, or PP1α and β siRNAs and then with auxin (2 h) or not. Graph shows RNA fold change at each amplicon relative to control siRNA transfected cells not treated with auxin following normalization to spliced ACTB. *n* = 3. Error bars are SEM.

Recent results in fission yeast implicate PP1 in reducing elongation rate after the PAS through its dephosphorylation of SPT5 and Pol II CTD (Kecman et al. 2018; Parua et al. 2018). We tested whether PP1 activity is involved in the Pol II slowing first using a small molecule inhibitor approach. This has possible advantages over protein depletion: It is more acute, may not disrupt PP1-associated complexes, and is more easily combined with XRN2 depletion to test the relationship between PP1 activity and torpedo termination in the same cells. We assessed the impact of PP1 on XRN2-dependent termination using Pol II ChIP on *ACTB* and *MYC* in *XRN2-AID* cells treated with auxin, the selective PP1 inhibitor, tautomycetin (Choy et al. 2017), or both (Fig. 5A). XRN2 loss showed the expected termination defect on both genes characterized by an accumulation of Pol II downstream from the PAS. PP1 inhibition gave a similar Pol II profile to the untreated control situation with a slight tendency for more readthrough transcription. When XRN2 was depleted from PP1 inhibited cells, the post-PAS accumulation of Pol II that we had characterized as resulting from accumulated slow polymerase was reduced. However, the readthrough at extended positions was maintained. These observations support the idea that PP1 facilitates XRN2-dependent termination by promoting Pol II slowing.

PP1-assisted Pol II slowing might facilitate termination by expediting the capture of polymerases by XRN2, which we showed to be an important part of the mechanism in Figure 2F. To interrogate this, total RNA was isolated from *XRN2-AID* cells following PP1 inhibition and/or XRN2 depletion and transcriptional readthrough was assayed for *ACTB* and *MYC* by qRT-PCR (Fig. 5B). As expected, XRN2 loss induced readthrough at both genes. Tautomycetin also induced transcriptional readthrough, suggesting that PP1 activity is important for efficient termination. Treatment with tautomycetin and auxin gave the biggest effect and was especially strong at positions furthest beyond the PAS. These effects are larger than in the above ChIP experiments. This is because ChIP signal derives from Pol II occupancy of a short fragment of DNA (due to prior sonication), whereas qRT-PCR signal derives from intact transcripts and reports everything over and beyond a tested amplicon.

Mass spectrometry identified PP1α and β isoforms as components of the 3′ end processing complex (Shi et al. 2009). We therefore specifically tested their involvement in transcriptional readthrough using RNAi to deplete either or both from *XRN2-AID* cells (Fig. 5C). We then used qRT-PCR to assay transcriptional readthrough under these conditions following treatment or not with auxin to deplete XRN2 (Fig. 5D). Depletion of PP1 by itself did not lead to extended readthrough; however, it did so when XRN2 was subsequently depleted. This was the case for both *MYC* and *ACTB* with the effect more subtle for the latter. Analysis of another gene (*YTFDH3*) confirmed that PP1 depletion enhances readthrough when XRN2 is eliminated. An additional protein phosphatase inhibitor, Calyculin A, was used to further confirm the protein phosphatase effect on extended *ACTB* readthrough (Supplemental Fig. S4). The combined interpretation of this ChIP and qRT-PCR is that PP1 is important for Pol II slowing after the PAS and for its efficient capture and termination by XRN2.

### Directed RNaseH1 activity bypasses the requirement for CPSF73 in transcriptional termination

**Figure 6.**
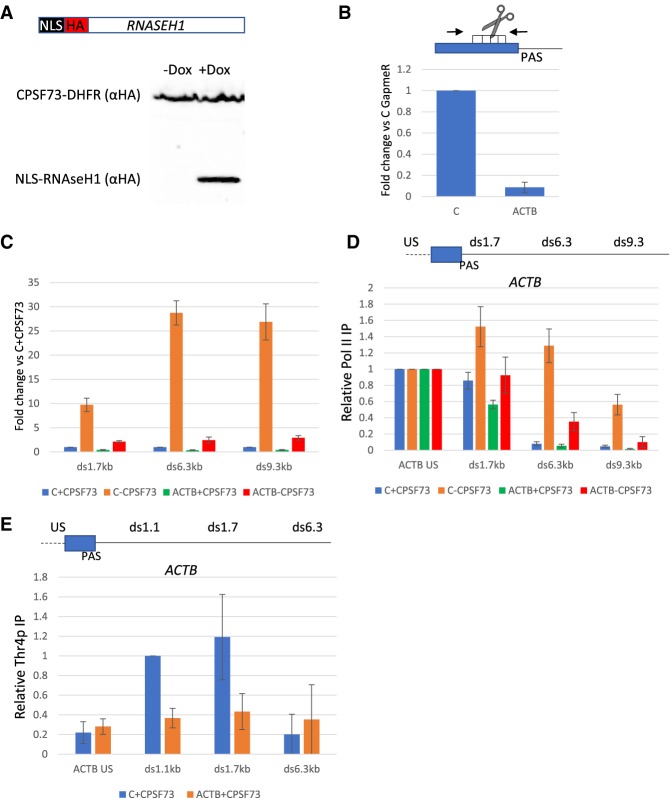
Directed RNaseH1 activity promotes transcriptional termination in the absence of CPSF73. (*A*) Western blot to detect NLS-RNaseH1 introduced into *CPSF73-DHFR* cells. Its expression is dox-dependent and CPSF73 is shown as a loading control. (*B*) qRT-PCR of NLS-*RNASEH1*-modified *CPSF73-DHFR* cells transfected with control or ACTB GapmeRs. Primers flanking the GapmeR targeting site were used to assess cleavage efficiency shown as a fold change in RNA levels relative to control GapmeR following normalization to spliced GAPDH. *n* = 4. Error bars are SEM. (*C*) qRT-PCR of RNaseH1-modified *CPSF73-DHFR* cells transfected with control or ACTB GapmeR and grown in the presence or absence of TMP (12 h). Graph shows fold change in RNA levels compared with control GapmeR transfected cells grown in the presence of TMP (C + CPSF73) following normalization to unspliced ACTB RNA. *n* = 3. Error bars are SEM. (*D*) Pol II ChIP of RNaseH1-modified *CPSF73-DHFR* cells transfected with control or ACTB GapmeRs and grown in the presence or absence of TMP (12 h). Graph shows relative Pol II IP normalized to Pol II occupancy upstream of the PAS (ACTB US) in each condition. *n* = 3. Error bars are SEM. (*E*) Thr4p ChIP of RNaseH1-modified *CPSF73-DHFR* cells transfected with control or ACTB GapmeRs. Graph shows relative Pol II IP normalized to occupancy over the ds1.1-kb amplicon in control GapmeR transfected cells grown in TMP (C + CPSF73). *n* = 3. Error bars are SEM.

The importance of XRN2 in transcriptional termination is shown by the genome-wide termination defect in its absence. PP1 may also play a direct active role in termination or it could serve to facilitate the XRN2-dependent process by slowing Pol II down. Disentangling these possibilities requires experiments to isolate allosteric and torpedo components from one another. To do this we tested whether alternative endoribonucleolytic cleavage could support termination on a protein-coding gene following CPSF73 loss, as this would potentially separate its RNA cleavage and allosteric functions. As the XRN2-incompatible RZ cleavage does not support termination in the absence of CPSF73 (Fig. 4A), we used RNaseH1, which cuts RNA:DNA hybrids and generates substrates for XRN2 (Hori et al. 2015). To concentrate high levels of RNaseH1 in the nucleus we integrated it into the genome after replacing its mitochondrial localization signal with a nuclear localization signal (to make *NLS-RNASEH1*). Because the requisite puromycin-resistance marker had been used in *CPS73-AID* cells (to introduce TIR1), dox-inducible *NLS-RNASEH1* was integrated into our previously described *CPSF73-DHFR* cell line (Eaton et al. 2018). In those cells, CPSF73 is depleted by removing trimethoprim (TMP) from growth media. The Western blot in Figure 6A confirms the successful introduction dox-inducible *NLS-RNASEH1*.

Next, we designed a GapmeR to the last exon of *ACTB* close to the PAS. GapmeRs are modified antisense oligonucleotides that, when bound to their target transcript, result in cleavage by RNaseH1. ACTB or control GapmeRs were transfected into *NLS-RNASEH1*-expressing *CPSF73-DHFR* cells. We then added dox in the presence or absence of TMP to retain or deplete CPSF73. qRT-PCR, using primers that could only detect uncleaved products, revealed very efficient (>90%) GapmeR-induced cleavage (Fig. 6B). GapmeR-directed cleavage also suppressed the strong readthrough caused by CPSF73 depletion as judged by the reduction in RNA levels beyond the *ACTB* PAS (Fig. 6C). This contrasts with the inability of RZ cleavage to do the same on *RBM3* (Fig. 4A), correlating these effects with the compatibility of cleaved RNA with 5′ → 3′ degradation. Crucially, Pol II ChIP confirmed that the large termination defect caused by CPSF73 depletion was almost completely suppressed by introducing the ACTB GapmeR (Fig. 6D). This experiment shows that CPSF73 itself is not a strict requirement for termination and that an important part of its normal function in the process is to cleave the transcript.

As RNaseH1 cleavage of *ACTB* RNA promotes termination in the absence of CPSF73, it is likely to do so without associated Pol II slowing. Based on our model, this means that RNaseH1 cleavage might be relatively faster than at the *ACTB* PAS to facilitate Pol II pursuit by XRN2. To test this assumption, we performed Thr4p ChIP in RNaseH1-expressing *CPSF73-DHFR* cells transfected with control or ACTB GapmeRs. Because Thr4p depends on CPSF73 (see Fig. 3B), its presence will indicate prior CPSF73 activity. In control treated cells, Thr4p was highest at 1.1 and 1.7 kb beyond the PAS as expected (Fig. 6E). However, Thr4p did not accumulate at those positions when the ACTB GapmeR was transfected, even though CPSF73 is still present. This suggests that GapmeR-directed cleavage is often faster than at the PAS and provides an explanation for how it promotes efficient termination even in the absence of CPSF73.

### RNA cleavage in different locations and by different RNases promotes transcriptional termination without CPSF73

**Figure 7.**
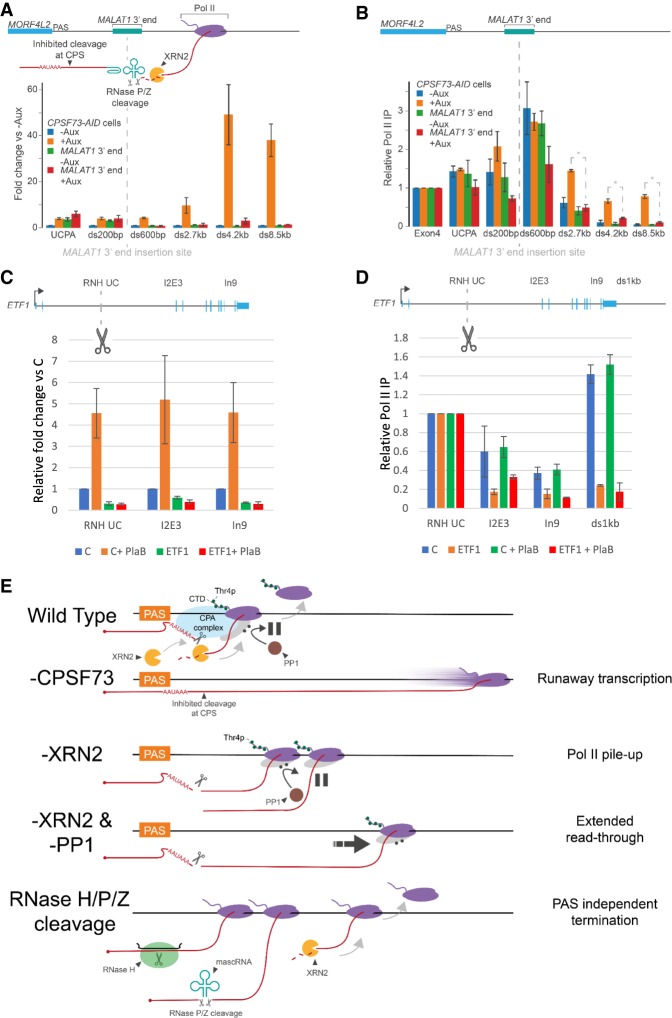
Other RNases promote termination in the absence of CPSF73, which can also occur within gene bodies. (*A*) qRT-PCR analysis of *MORF4L2* readthrough in unmodified *CPSF73-AID* cells and *CPSF73-AID* cells modified at *MORF4L2* by inserting the *MALAT1* 3′ end and then treated or not with auxin (3 h). The graph shows fold change in RNA at each amplicon relative to unmodified CPSF73*-AID* cells not treated with auxin after normalizing to spliced ACTB. *n* = 3. Error bars are SEM. UCPA detects transcripts not cleaved at the PAS. (*B*) Pol II ChIP analysis of *MORF4L2* in unmodified *CPSF73-AID* cells and CPSF73*-AID* cells modified at *MORF4L2* by inserting the *MALAT1* 3′ end and then treated or not with auxin (3 h). The graph shows relative Pol II IP normalized to occupancy over *MORF4L2* exon 4 in each case. *n* = 3. Error bars are SEM. (*C*) qRT-PCR analysis of *ETF1* GapmeR cleavage effects in RNaseH1-modified *CPSF73-DHFR* cells transfected with control or ETF1 GapmeRs, grown in the presence of TMP before treatment or not with PlaB (3 h). The graph shows fold change in RNA at each amplicon relative to control GapmeR-treated cells grown without PlaB after normalizing to spliced ACTB. *n* = 3. Error bars are SEM. GapmeR cleavage site is illustrated (scissors) in the schematic. (*D*) Pol II ChIP of RNaseH1-modified *CPSF73-DHFR* cells transfected with control or ETF1 GapmeRs and grown in the presence of TMP before treatment or not with PlaB (3 h). Graph shows relative Pol II IP normalized to occupancy over the GapmeR target site in each case. *n* = 3. Error bars are SEM. (*E*) Model. Normally, Pol II pauses beyond the PAS in a manner requiring PP1 activity and associated with Thr4p, which facilitates its pursuit and termination by XRN2. (-CPSF73) Pol II engages in runaway readthrough transcription; (-XRN2) paused Pol II is paused over 3′ flanking regions unable to terminate efficiently; (-XRN2 and PP1) this situation shows extended readthrough relative to loss of XRN2 alone, which is associated with reduced pausing after the PAS; (RNaseH/P/Z cleavage) bypasses of CPSF73/PAS requirement by promoting Pol II termination in association with 5′ → 3′ RNA degradation. Filled dots represent phosphates.

*NEAT1* and *MALAT1* noncoding genes terminate via CPSF73-independent yet XRN2-dependent mechanisms (Supplemental Fig. S5), perhaps because their RNAs undergo 3′ end cleavage by RNaseP/Z (Wilusz et al. 2008). We used CRISPR–Cas9 to insert the 3′ end processing element from *MALAT1* into the 3′ flank of *MORF4L2* in *CPSF73-AID* cells. We performed qRT-PCR to assay *MORF4L2* readthrough in modified and unmodified *CPSF73-AID* cells treated or not with auxin (Fig. 7A). While CPSF73 loss induced very strong readthrough at the unmodified *MORF4L2,* this effect was suppressed in the presence of the *MALAT1* 3′ end, strongly suggesting that RNaseP/Z bypasses the need for CPSF73. Interestingly, the MALAT1 3′ end also caused an increase in the level of *MORF4L2* transcripts that were not cleaved at the PAS, even when CPSF73 is present. This argues that RNaseP/Z cleavage often occurs before/without cleavage at the upstream PAS similar to GapmeR-directed RNaseH1 at *ACTB*. ChIP analysis confirmed that the MALAT1 3′ end suppresses the effects of CPSF73 loss on *MORF4L2* termination (Fig. 7B). Thus, alternative (and endogenous) RNases can promote termination on protein-coding genes in the absence of CPSF73.

As these directed RNase experiments occur close to a PAS, there might be other PAS-dependent features that promote termination even without CPSF73. To see whether this is the case, we designed a GapmeR to cleave the second intron of *ETF1* far upstream of its PAS. Importantly, *ETF1* is unlikely to contain cryptic PAS elements since its transcripts do not undergo premature CPA when U1 snRNA is inhibited (Kaida et al. 2010). To test the potential for *ETF1* intron cleavage to promote premature transcriptional termination, we performed qRT-PCR in *NLS-RNASEH1* expressing *CPSF73-DHFR* cells (without depleting CPSF73) transfected with control or ETF1 GapmeRs (Fig. 7C). We also analyzed equivalent samples following splicing inhibition using Pladienolide B (PlaB) in case intron removal confounded pre-mRNA detection. Indeed, PlaB enhanced all three intron-derived species; however, this was almost fully suppressed by the ETF1 GapmeR. This included over the cleavage site (showing efficient cutting) and at downstream position. Pol II ChIP confirmed that the ETF1 GapmeR induced premature termination of transcription and did so in the presence and absence of splicing (Fig. 7D). These experiments indicate that RNA degradation is, at least in principle, sufficient for termination, with allosteric features used to facilitate it in practice. While a PAS-induced modification is not strictly necessary for transcriptional termination, it is probably required for the XRN2-dependent process to be most efficient.

## Discussion

We have shown that rapid CPSF73 depletion causes runaway readthrough, which we propose is because processes underpinning transcriptional termination are lost. We identified allosteric and torpedo elements of the transcriptional termination mechanism that were enriched by rapid depletion of XRN2. Our experiments reveal the following mechanism: CPSF73 function (and likely PAS cleavage) promotes Thr4p with PP1 activity playing an important role in slowing Pol II down to facilitate its capture by XRN2. The allosteric events most likely aid XRN2 rather than acting as a separate or mutually exclusive termination pathway because cleavage of primary transcripts promotes termination in the absence of CPSF73 and outside of a PAS context. Consequently, we propose that the long-standing allosteric and torpedo models for transcriptional termination can be unified into a single mechanism with XRN2 likely providing the termination activity (Fig. 7E).

This conclusion followed from our failure to detect efficient/general XRN2-independent termination, although we did observe some evidence for this in two (*MYC* and *TRIB1*) cases. However, 3′-flanking transcripts were always much more sensitive to XRN2 loss than to exosome depletion and remained largely chromatin-associated under all circumstances. While there may be inefficient or stochastic XRN2-independent termination, it does not seem to be linked by a common mechanism. We do not rule out XRN2-independent termination that evades our detection or that occurs on certain genes, but our subsequent finding that PP1 inhibition causes longer readthrough when XRN2 is depleted argues against efficient alternatives.

Our results suggest that endoribonucleolytic cleavage might be an elemental function of CPSF73 in termination. However, polymerases display runaway readthrough in the absence of CPSF73 and are not modified by Thr4p, suggesting that its absence also prevents allosteric events. There are two broad explanations for this: The first is that CPSF73 loss prevents the assembly of other 3′ end processing factors that also promote allostery, and the second is that allosteric events occur downstream from PAS cleavage. While not mutually exclusive, we favor the latter because of our previous finding that inactive CPSF73 does not support termination (Eaton et al. 2018). Furthermore, PP1 depletion does not affect PAS cleavage (Shi et al. 2009), but still causes extended transcriptional readthrough.

Part of the mechanism for how PP1 activity causes Pol II to slow down is explained by recent data from fission yeast, in which dephosphorylation of SPT5 occurs specifically beyond the PAS (Parua et al. 2018). Consistently, and while we were revising this manuscript, the Bentley lab demonstrated dephosphorylation of SPT5 beyond the PAS in human cells associated with XRN2-dependent termination (Cortazar et al. 2019). Even so, other relevant factors may be subject to PP1 activity because phosphomimetic mutations in SPT5 do not lead to termination defects in fission yeast (Kecman et al. 2018). For instance, p54nrb is among the many PP1-interacting proteins and promotes the recruitment of XRN2 to genes (Kaneko et al. 2007; Heroes et al. 2013). Elucidating the complete network of phospho-regulation during transcriptional termination is an interesting and extensive area for future study.

The importance of Pol II slowing for XRN2-dependent termination likely relates to the respective rates of RNA transcription and degradation. Pol II elongation rate is on average ∼2 kb/min with some variation on different genes (Singh and Padgett 2009; Fuchs et al. 2014). Although the rate of XRN2 degradation is unknown, the closely related 5′ → 3′ exonuclease, XRN1, has recently been measured at ∼2 kb/min (Hoek et al. 2019). Assuming that XRN2 behaves similarly, rates of synthesis and degradation are closely matched. Without pausing, XRN2 may be unable to catch the polymerase where PAS cleavage is slow or when Pol II elongation is faster than the average rate. In support of closely matching rates, increasing Pol II elongation rate by only 220 nt/min induces a substantial downstream shift of termination positions (Fong et al. 2015).

Finally, the GapmeR and RNase P/Z experiments show that CPSF73-dependent events can be bypassed by rapid cleavage. This result also implies that the elemental role of CPSF73 (and the PAS) in termination is to provide an end for XRN2, otherwise alternative cleavage would not function in its absence. It also demonstrates that CPSF73-dependent changes occurring beyond the PAS facilitate termination (we propose, by slowing down Pol II) rather than directly contributing. This is consistent with the ability for XRN2 to promote termination in vitro in the absence of other factors (Park et al. 2015). This experiment also has additional implications for the study of noncoding RNA function, which often relies on the assumption that their targeting by antisense oligonucleotides does not affect transcription of their locus. We suggest that premature transcriptional termination might also occur in some of these experiments.

## Materials and methods

### Cell culture and transfections

HCT116 cells were cultured in DMEM (high glucose) containing penicillin/streptomycin and 10% FBS at 37°C. DNA transfections were done with Jetprime (polyplus) and RNAi with Lipofectamine RNAiMAX (Life Technologies) following the manufacturers’ guidelines. Two siRNA transfections were used: the second 24 h after the first and RNA isolation 48 h after that. PP1 RNAi was performed using a single siRNA transfection with RNA isolated 72 h later. GapmeRs were transfected at 10 nM in 24-well or 100-mm dishes for RNA and ChIP experiments, respectively. This was done using 1.5 or 10 μL of Lipofectamine RNAiMAX in a final combined volume of 50 or 500 μL of OptiMEM (Life Technologies). Dox was used at 1 μg/mL, auxin was used at 500 μM, tautomycetin was used at 500 nM, PlaB was used at 1 nM, and calyculin A was used at 5 nM. TMP was used at 20 μM and withdrawn for 12 h to deplete CPSF73-DHFR.

### Cell lines and cloning

The *XRN2-AID* and *CPSF73-eDHFR* cell lines are previously described (Eaton et al. 2018). *NLS-RNASEH1* was synthesized by Integrated DNA Technologies and cloned into pMK243 (Addgene 72835) digested with MluI and BglII. This plasmid was transfected with AAVS1 T2 CRISPR plasmid (Addgene 72833), and colonies were selected in 1 μg/mL puromycin. The *CPSF73-AID* cell line was made by generating plasmids containing a 5′ homology arm – AID – P2A – HYG/NEO – SV40 PAS – 3′ homology arm. We have published the sequences of each of these elements (Eaton et al. 2018). These, together with px330 (Addgene 42230) containing a CPSF73 guide RNA sequence, were transfected into HCT116 cells containing dox-inducible TIR1 and colonies selected with 30 μg/mL hygromycin and 800 μg/mL neomycin. HCT116 cells containing inducible TIR1 were made by transfecting HCT116 cells with pMK243 and AAVS1 T2 CRISPR plasmid, followed by selection in 1 μg/mL Puromycin. The *RBM3* and *MORF4L2* gene insertions were made using coselection (Agudelo et al. 2017). gRNA sequences were cloned into Addgene plasmid 86611 and transfected with a plasmid containing insertion elements (see the Supplemental Material) flanked with homology arms. Positive clones were selected by growth in 0.5 μM ouabain. Insertions were confirmed by Sanger sequencing.

### Antibodies

The following antibodies were used: CPSF73 (Bethyl Laboratories A301-090A), Tubulin (Abcam Ab7921), HA (Roche 3F10), Thr4p (Active Motif 6D7), EXOSC10 (Santa Cruz Biotechnology Sc-374595-X), PP1α (Bethyl Laboratories A300-904A), PP1β (Bethyl Laboratories A300-905A), and RNA Pol II (MBL Technologies CMA601 and Abcam 8WG16). Note that CMA601 was discontinued during our study and used in Figures 3A and 6D.

### Total RNA isolation and qRT-PCR

RNA was isolated using tri-reagent and, following DNase treatment, 1 μg was reverse transcribed (Protoscript II, New England Biolabs) with random hexamers. cDNA was diluted to 50 μL in water and 1 μL was used per qPCR, performed with LUNA qPCR reagent (New England Biolabs) in a Qiagen Rotorgene instrument.

### Chromatin-associated and nucleoplasmic RNA isolation

A six-well dish of cells was lysed in in HLB (10 mM Tris pH 7.5, 10 mM NaCl, 2.5 mM MgCl_2_, 0.5% NP40); this was underlayered with HLB + 10% sucrose and spun at 500×*g* for 5 min. Nuclei were resuspended in 100 μL of NUN1 buffer (20 mM Tris.HCl pH 7.9, 75 mM NaCl, 0.5 mM EDTA, 50% glycerol, 0.85 mM DTT) and then topped up with 1 mL of NUN2 (20 mM HEPES pH 7.6, 1 mM DTT, 7.5 mM MgCl_2_, 0.2 mM EDTA. 0.3 M NaCl, 1 M urea, 1% NP40). After incubation for 10 min on ice with mixing every 2–3 min, samples were spun at 13,000 rpm for 10 min. Chromatin pellets were resuspended in 500 μL of Tri-reagent and RNA was isolated with Trizol as above. Nucleoplsamic RNA was extracted from the supernatant by phenol chloroform and ethanol precipitation.

### RNA sequencing

For chromatin-associated RNA-seq, cells were treated with dox/EtOH (18 h) and then with auxin/EtOH (3 h). Nuclear RNA-seq was performed following treatment with dox (18 h) and then auxin/EtOH (3 h). Five-hundred nanograms was rRNA depleted using the Ribozero kit, and libraries were prepared with the tru-seq stranded kit and sequenced on an illumina Hi-seq 2500. Data processing steps are described in the Supplemental Material.

### Chromatin immunoprecipitation

A semi-confluent 100-mm dish of cells was cross-linked in 1% fomaldehyde for 10 min before quenching in 125 mM glycine. Cells were pelleted and resuspended in 350 μL RIPA buffer (150 mM NaCl, 1% NP40, 0.5% sodium deoxycholate, 0.1% SDS, 50 mM Tris.HCl pH 8, 5 mM EDTA pH 8) before sonication in a Bioruptor for 30 sec on and 30 sec off 10 times on high. Cross-linked chromatin was divided into two: Half was incubated with 40 μL of sheep antimouse/sheep antirat Dynabeads (Life Technologies) preincubated with 3 μg of Pol II/Thr4p antibody for 2 h at 4°C, and the other half with beads incubated without antibody. Following rotation for 3 h at 4°C, beads were washed twice in RIPA buffer, three times in ChIP wash buffer (500 mM NaCl, 1% NP40, 1% sodium deoxycholate, 100 mM Tris.HCl pH 8.5), and twice in RIPA buffer (ChIP wash washes were not used for Thr4p). DNA was eluted in 0.1 M NaHCO3/1% SDS with rotation for 30 min at room temperature. Eluate was added to 30 μL of 5 M NaCl and cross-links were reversed overnight at 70°C. DNA was purified by phenol chloroform extraction and ethanol precipitation and resuspended in 50 μL of water. One microliter was used per qPCR.

### Gene expression omnibus accession numbers

RNA-seq and mNET-seq of *XRN2-AID* cells (GSE109003); chromatin and nuclear RNA-seq of *CPSF73-AID* cells (GSE137727); CPSF73 RNAi chromatin RNA-seq (GSE60358).

### DNA sequences

All DNA sequences are provided in the Supplemental Material.

## Supplementary Material

Supplemental Material
