# Supplementary material for: A unified allosteric/torpedo mechanism for transcriptional termination on human protein-coding genes
Source: Genes Dev. 2020 Jan 1;34(1-2):132–45. doi: 10.1101/gad.332833.119 (PMC6938672; doi:10.1101/gad.332833.119)
Supplement: Supplemental Material [file supp_34_1-2_132__index.html]

A unified allosteric/torpedo mechanism for transcriptional termination on human protein-coding genes — Supplemental Material 

# A unified allosteric/torpedo mechanism for transcriptional termination on human protein-coding genes

## Supplemental Material

- Supplemental\_Methods.pdf
- SUPPLEMENTAL\_FIGS.pdf
