# Supplementary material for: A unified allosteric/torpedo mechanism for transcriptional termination on human protein-coding genes
Source: Genes Dev. 2020 Jan 1;34(1-2):132–45. doi: 10.1101/gad.332833.119 (PMC6938672; doi:10.1101/gad.332833.119)
Supplement: Supplemental Material [file supp_gad.332833.119_Supplemental_Methods.pdf]

## Supplemental Methods

### 4sU RNA isolation

Cells were incubated in 500uM 4-thiouridine for 2.5 mins before RNA extraction using Trizol. 15-20ug RNA was biotinylated in a volume of 250µl containing 10mM HEPES (pH7.5), 5ug MTSEA Biotin-XX (Iris Biotech, dissolved in dimethyl formamide). After incubation in the dark for 90 mins, biotinylated RNA was chloroform extracted, phenol chloroform extracted and ethanol precipitated. It was re-suspended in RPB (300mM NaCl, 10mM Tris pH7.5, 5mM EDTA) and incubated with 50ul streptavidin-coated magnetic beads (Miltenyi Biotech) for 15 mins. Beads were washed 5x in (100 mM Tris-HCl pH 7.4, 10 mM EDTA, 1 M NaCl, and 0.1% Tween-20) pre-heated to 60$^{o}$C. RNA was eluted in 100µl of 0.1M DTT for 15 mins at 37$^{o}$C before final phenol chloroform extraction and ethanol precipitation.

### Metaprofiling of effects of XRN2 vs CPSF73 depletion (Figure 2A)

Single-end 50-base-pair (bp) reads were screened for sequencing quality using FastQC (http://www.bioinformatics.babraham.ac.uk/projects/fastqc); adapter sequences were then removed using Trim Galore using the default settings (https://www.bioinformatics.babraham.ac.uk/projects/trim_galore). Trimmed reads passing QC steps were aligned to the GRCh38 (Ensembl) human genome using Hisat2 with default parameters, incorporating known splice sites (Kim et al. 2015). Unmapped, multi-mapped and reads with low mapping quality scores (MAPQ <20) were discarded using SAMtools (Li et al. 2009).

For metagene analysis, expressed protein-coding genes (>50 reads per gene) were selected and a window extending 20 kb downstream from the transcription end site (TES) was applied. Overlapping gene intervals including genes that extend beyond the chromosome boundaries were, regardless of expression, discarded using the BEDtools suite (Quinlan and Hall 2010), leaving 625 usable protein-coding genes for metagene analysis. Read-coverage over these intervals was normalised to reads-per-kb-per-million (RPKM) using deeptools. Further graphical processing of expression profiles was then performed within the R environment (http://www.R-project.org).

### Chromatin associated RNA-seq processing

For chromatin-associated RNA-seq raw 50bp single-end reads were assessed for quality using FASTQC followed by adapter sequence and quality trimming using TrimGalore!, where reads shorter than 20bp were discarded. Reads were aligned to human GRCh38.p10 using Hisat2 with known splice sites extracted from Gencode release 27 (Kim et al. 2015). Primary mapped alignments were strand-separated using SAMtools before normalised bigWig

files were generated using the deepTools with BPM (equivalent to TPM for RNA-seq samples) normalisation option (Li et al. 2009; Ramirez et al. 2016). Gene and chromosome snapshots were generated with deepTools/pyGenomeTacks package using bigWig and Gencode v27 annotation files with only a primary isoform shown for clarity (Ramirez et al. 2018). The 100kb chromatin RNA-seq metagene was generated using primary transcript isoforms of expressed genes >100kb from expressed neighbouring genes (determined in minus auxin conditions) using a custom script. The output matrix data from deepTools was used to plot the graph using Plotly.

**Nucleic acid sequences**

*Gapmers*

ACTB: TAAGGCGAAGATTAA

ETF1: GAATAGATTCATGCTG and AAGTAGTGGGCCATCT

Control (targets to *HBB* locus, which is not expressed in HCT116): ATAGAAATTGGACAGC

*Oligonucleotides for qPCR*

ACTB US: tcaaggtgggtgtctttcct/cctgcttgctgatccacatc
ACTB ds1.1kb: tgccttccctctgctagaag/tgtgcacagttgagagtcca
ACTB ds1.7kb: ccaaccagatgtgttccgtg/caagaccaccaccacaatcg
ACTB ds6.3kb: aggaggcaatgctggagaat/gtacctgggaactctgcact
ACTB ds9.3kb: cagggaagacgtgctaggaa/tcctttctcctctgctcagc
Myc US: attacaggtgtgagccaggg/agcctgcctcttttccaca
Myc UCPA: atcattgagccaaatcttaagttgtg/ctctgaaggggcaattgatga
MYC ds1.2kb: ctcctgggaagaagccagtt/gggccataggttttcagagg
MYC ds1.8kb: ggcgctcttaaacagctcag/ccaagctccacatccctaaa
MYC ds2.5kb: aaatgccgagggatgttctc/tctcctgacctcatgatccg
MYC ds5kb: tggaagaggagccaaaggag/ggaagctgcgttcatgtgat
MYC ds7.6kb: gaacccctctttccctccaa/ccccaaagctaccacaggat
MYC ds15kb: tgggaaaggggcagttgta/atggtggggcattctctgaa
RPL30 flank: actgcactgggttcctttct/tgacagcattttggatgggg
EIF3E flank: tggctcctcagtcactcac/ggctgtcagttcaaccaaagt
EIF3H flank: tggatggattggaagagggg/caaacaggctcaaggtgcat
TRIB1 flank: agaaacgtttgaaggtgagca/ggttcacaatcggaagtccc
RBM39 PROMPT: ggaaatagtggagaaaagca/catttttgaaggaacggtag
MORF4L2 Ex4: tcttgaaccagctctcccag/tactgccaccatctccgttt
MORF4L2 UCPA: gtagccacggttttctggaaa/ accagtaacatgaaaggcacac
MORF4L2 ds200: tgttactggttggtattctggt/ tttgagtcccatttatttgctgg
MORF4L2 ds600: accccagtgacctcatttagt/ acacccgccaaattcatgtt
MORF4L2 ds2.7kb: agcatgctagtgggaaatcc/ggatctcctcaggctttggt
MORF4L2 ds4.2kb: ccccatgacattcagtgcct/tgcttccgtaccaatccaca
MORF4L2 ds8.5kb: gccaaggacacacagctaag/ tccttttcagagagccagga
MORF4L2 ds20kb: gtggaaatcgaggcagcaat/ gactgacctgtgttggcaac
YTHDF3 ds10kb: acaaaaggacagcagaggga/agcctcttcttatgccaccc
YTHDF3 ds20kb: agcagctgtctagacccaag/gtagcaacgcctttccagag

RBM3 UCPA: tgctgtgaaagagtatattcgt/gtctgccttgtttcttggctcc
RBM3 ds1.1kb: gaatcaggcatttacaggactggc/agcgcatgcccaattaccttttac
RBM3 ds8.5kb: ccattgtggtcagaaaggctcttg/tggaccccaccaatgcatgatata
RBM3 ds11kb: gggcagtaaacccctctagagttc/ggttgtggtgatagcctgcattac
ETF1 RNH UC: acactgttcctctcatggca/aggtggggcatatggaatgt
ETF1 I2E3: gcatctccctgtagcttgct/gccactcgtgaaatctggtc
ETF1 IN9: gtcttgctgtgttcaccagg/aggtttcacagtgcaggaga
ETF1 ds1kb: ttccctcctaatgcgtgtct/tctcagccctaaatccaccg

*Oligos for cloning guide sequences*

RBM3: caccgttatctatgataactagca/aaactgctagttatcatagataac
MORF4L2: caccgtccctgagttgccaccagag/aaacctctggtggcaactcagggac

*siRNAs*

Control: silencer select siRNA negative control
EXOSC3: Thermo Fisher Silencer Select siRNA inventory#: s532991
EXOSC10: Thermo Fisher Silencer Select siRNA inventory#: s10738
PP1α: Thermo Fisher Silencer Select siRNA inventory#: s10930
PP1β: Thermo Fisher Silencer Select siRNA inventory#: s10935

*Other DNA sequences*

*δRZ[WT]*
AGGGCGGCATGGTCCCAGCCTCCTCGCTGGCGCCGCCTGGGCAACATGCTTCGGCATGGCG
AATGGGACCAAA

*δRZ[MT]*
AGGGCGGCATGGTCCCAGCCTCCTCGCTGGCGCCGCCTGGGCAACATGCTTCGGCATGGTG
AATGGGACCAAA

*xrRNA*
GTACTTCGAAATGTCATCCTCTGTCTGACACTGAACGTAATCCAGACGCGTAAGTCAGG
CCGGAAAATTCCCGCCACCGGAAGTTGAGTAGACGGTGCTGCCTGCGACTCAACCCCA
GGAGGACTGGGTGAACAAAGCTGCGAAGTGATCCATGTAAGCCCTCAGAACCGTCTCG
GAAAGAGGACCCCACATGTTGTAGCTTCAAGGCCCAATGTCAGACCACGCCATGGCGT
GCCACTCTGCGGAGAGTGCAGTCTGCGACAGTGCCCCAGGAGGACTGGGTGAGGATC
CTACCTACAAACGGCACGAGCATCAGCC

*NLS-RNASEH1*
ATGCCCAAGAAGAAGCGCAAGGTGGGAGGCTATCCCTATGATGTACCAGATTACGCTG
GCGGAATGTTTTATGCCGTGAGGCGCGGTCGAAAGACAGGAGTGTTCCTGACCTGGAA
CGAATGTCGGGCACAAGTCGACCGCTTCCCTGCCGCGCGATTCAAGAAGTTCGCTACG
GAGGACGAAGCATGGGCATTTGTCCGCAAATCAGCATCTCCCGAAGTTTCAGAAGGTC
ACGAGAATCAGCACGGACAAGAAAGCGAGGCCAAAGCATCAAAGCGGCTCAGGGAGC
CTCTGGATGGTGATGGCCACGAATCAGCGGAGCCATATGCTAAACACATGAAACCGTC
TGTGGAACCAGCGCCCCCGGTTAGCAGAGACACTTTCTCCTATATGGGGGATTTCGTG
GTGGTTTATACGGACGGTTGTTGCTCTAGTAATGGACGACGGCGACCGCGAGCAGGTA
TAGGTGTCTACTGGGGGCCTGGGCACCCCCTGAATGTTGGGATTCGCCTTCCTGGGC

GGCAAACTAACCAGAGAGCAGAAATTCACGCAGCGTGTAAGGCGATTGAGCAAGCCAA
GACACAAAACATTAACAAGCTTGTACTTTACACTGATTCTATGTTCACGATCAACGGCAT
CACGAATTGGGTTCAAGGATGGAAAAAAAACGGCTGGAAAACCTCCGCAGGGAAGGAG
GTAATCAATAAAGAGGACTTTGTGGCTCTGGAAAGGCTTACTCAAGGAATGGACATTCA
ATGGATGCACGTGCCTGGACATTCCGGATTTATTGGAAATGAGGAGGCGGACCGATTG
GCTAGAGAGGGCGCAAAACAATCCGAAGATTAA

*MALAT1 3' end*

GGCCATGCAGGCCAATGCTCTTCAGTAGGGTCATGAAGGTTTTTCTTTTCCTGAGAAAA
CAACACGTATTGTTTTCTCAGGTTTTGCTTTTTGGCCTTTTTCTAGCTTAAAAAAAAAAAA
AGCAAAAGATGCTGGTGGTTGGCACTCCTGGTTTCCAGGACGGGGTTCAAATCCCTGC
GGCGTCTTTGCTTGGCCCTGAAGGCC

## REFERENCES


Kim D, Langmead B, Salzberg SL. 2015. HISAT: a fast spliced aligner with low memory requirements. *Nat Methods* **12**: 357-360.

Li H, Handsaker B, Wysoker A, Fennell T, Ruan J, Homer N, Marth G, Abecasis G, Durbin R, Genome Project Data Processing S. 2009. The Sequence Alignment/Map format and SAMtools. *Bioinformatics* **25**: 2078-2079.

Quinlan AR, Hall IM. 2010. BEDTools: a flexible suite of utilities for comparing genomic features. *Bioinformatics* **26**: 841-842.

Ramirez F, Bhardwaj V, Arrigoni L, Lam KC, Gruning BA, Villaveces J, Habermann B, Akhtar A, Manke T. 2018. High-resolution TADs reveal DNA sequences underlying genome organization in flies. *Nat Commun* **9**: 189.

Ramirez F, Ryan DP, Gruning B, Bhardwaj V, Kilpert F, Richter AS, Heyne S, Dundar F, Manke T. 2016. deepTools2: a next generation web server for deep-sequencing data analysis. *Nucleic acids research* **44**: W160-165.
