# Supplementary material for: A unified allosteric/torpedo mechanism for transcriptional termination on human protein-coding genes
Source: Genes Dev. 2020 Jan 1;34(1-2):132–45. doi: 10.1101/gad.332833.119 (PMC6938672; doi:10.1101/gad.332833.119)
Supplement: Supplemental Material [file supp_gad.332833.119_SUPPLEMENTAL_FIGS.pdf]

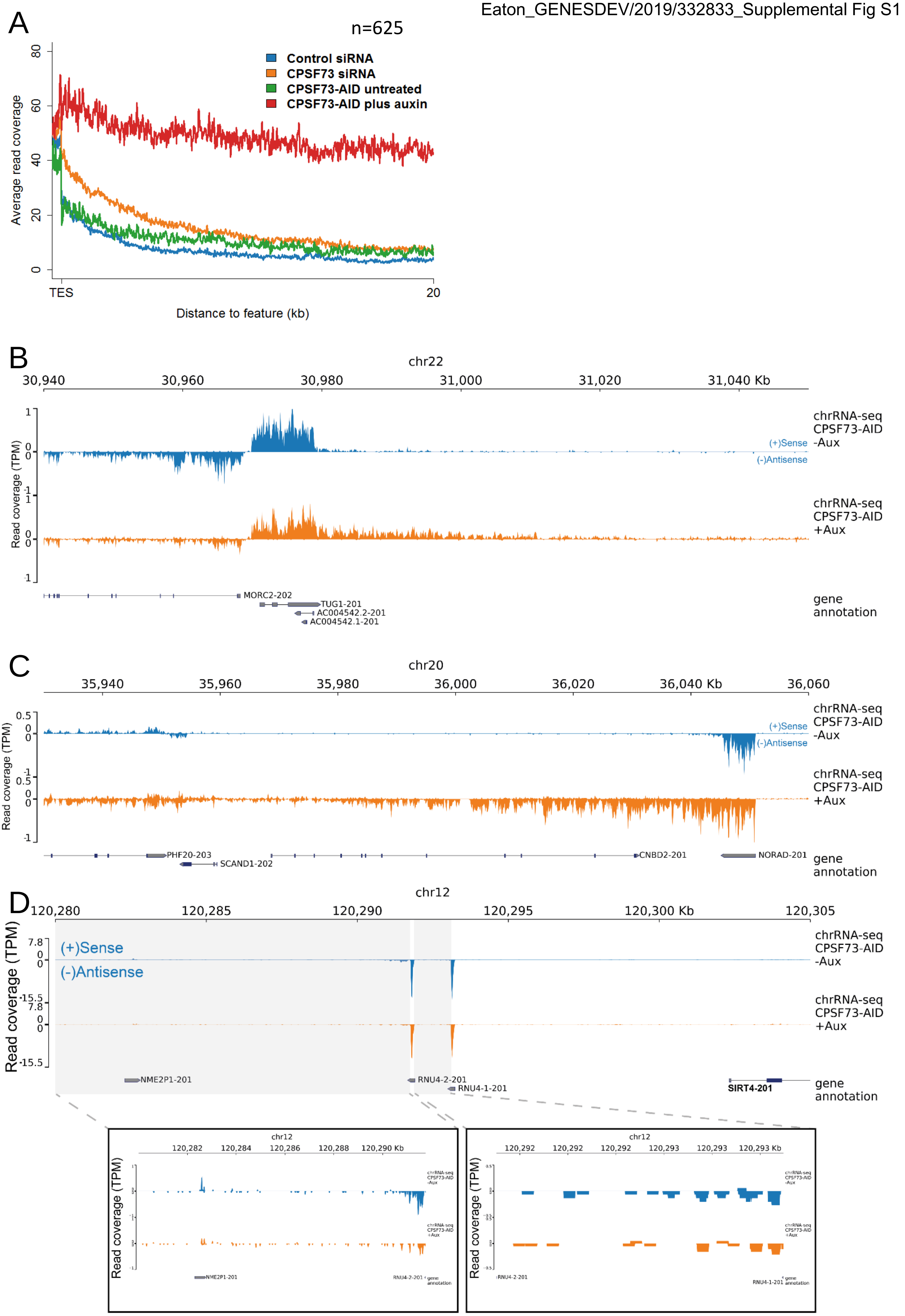
A
n=625
Control siRNA
CPSF73 siRNA
CPSF73-AID untreated
CPSF73-AID plus auxin
Average read coverage
Distance to feature (kb)
TES
20
B
chr22
30,940
30,960
30,980
31,000
31,020
31,040 Kb
chrRNA-seq CPSF73-AID -Aux
chrRNA-seq CPSF73-AID +Aux
(+)Sense
(-)Antisense
Read coverage (TPM)
gene annotation
MORC2-202
TUG1-201
AC004542.2-201
AC004542.1-201
C
chr20
35,940
35,960
35,980
36,000
36,020
36,040 Kb
36,060
PHF20-203
SCAND1-202
CNBD2-201
NORAD-201
D
chr12
120,280
120,285
120,290
120,295
120,300 Kb
120,305
NME2P1-201
RNU4-2-201
RNU4-1-201
SIRT4-201

Supplementary Fig. S1: Rapid depletion of CPSF73-AID produces much stronger transcriptional read-through than its depletion by RNAi

A: Metagene comparison of transcriptional read-through in chromatin RNA-seq samples obtained after CPSF73 RNAi (from Nojima et al., Cell 2015) versus rapid depletion of CPSF73-AID. Expressed genes were selected that were separated from one another by at least 20kb. Signals beyond the TES are normalised to gene body signal.

B: Chromosomal snapshot derived from chromatin RNA-seq of *CPSF73-AID* cells treated or not with auxin (3h) showing ncRNA *TUG1*. CPSF73 depletion reveals *TUG1* read-through that has previously been undetected using RNAi.

C: As in B, but for ncRNA *NORAD*.

D: Chromosomal snapshot derived from chromatin RNA-seq of *CPSF73-AID* cells treated or not with auxin (3h) showing *RNU4-1* and *RNAU4-2* snRNA genes which are unaffected by CPSF73 depletion.

A

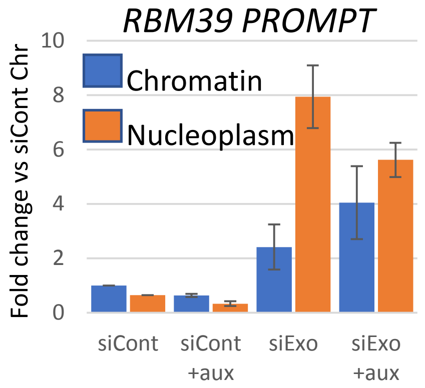
RBM39 PROMPT
Chromatin
Nucleoplasm
Fold change vs siCont Chr
0
2
4
6
8
10
siCont
siCont +aux
siExo
siExo +aux

B

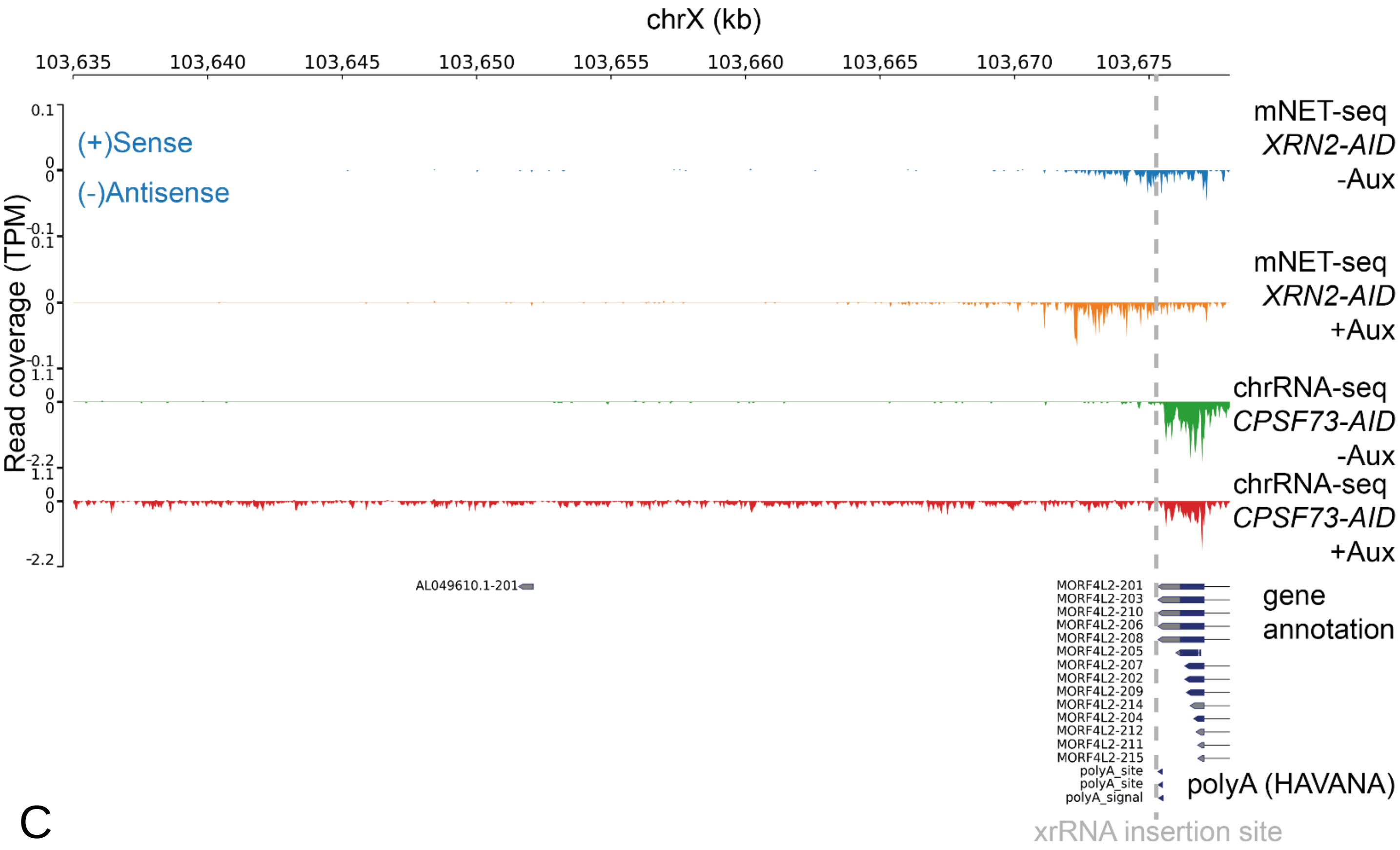
chrX (kb)
103,635
103,640
103,645
103,650
103,655
103,660
103,665
103,670
103,675
(+)Sense
(-)Antisense
Read coverage (TPM)
mNET-seq XRN2-AID -Aux
mNET-seq XRN2-AID +Aux
chrRNA-seq CPSF73-AID -Aux
chrRNA-seq CPSF73-AID +Aux
AL049610.1-201
MORF4L2-201
MORF4L2-203
MORF4L2-210
MORF4L2-206
MORF4L2-208
MORF4L2-205
MORF4L2-207
MORF4L2-202
MORF4L2-209
MORF4L2-214
MORF4L2-204
MORF4L2-212
MORF4L2-211
MORF4L2-215
polyA_site
polyA_site
polyA_signal
gene annotation
polyA (HAVANA)
xrRNA insertion site

C

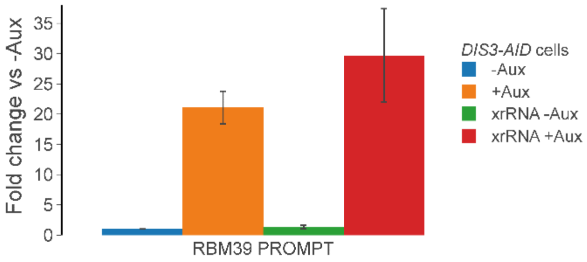
Fold change vs -Aux
0
5
10
15
20
25
30
35
RBM39 PROMPT
DIS3-AID cells
-Aux
+Aux
xrRNA -Aux
xrRNA +Aux

Supplemental Fig. S2: Rapid depletion of CPSF73-AID produces much stronger transcriptional read-through than its depletion by RNAi

A: qRT-PCR analysis of *RBM39* PROMPT in chromatin-associated or nucleoplamic RNA isolated from *XRN2-AID* cells transfected with control or EXOSC3/10 siRNAs before treatment or not with auxin (2h). RNA levels were quantitated relative to those found in chromatin-associated RNA from control siRNA transfected cells not treated with auxin (given a value of 1). n=3. Error bars are SEM.

B: Chromosomal snapshot of *MORF4L2* derived from chromatin RNA-seq of *CPSF73-AID* cells treated or not with auxin (3h) and from mNET-seq of *XRN2-AID* cells treated or not with auxin (2h). Grey dotted line shows xrRNA insertion site.

C: qRT-PCR analysis of *RBM39* PROMPT in unmodified *DIS3-AID* cells and *DIS3-AID* cells modified at *MORF4L2* by addition of an xrRNA and then treated or not with auxin (2h). The graph shows fold change in RNA relative to that found in unmodified *DIS3-AID* cells not treated with auxin after normalising to spliced ACTB. n=3. Error bars are SEM.

A

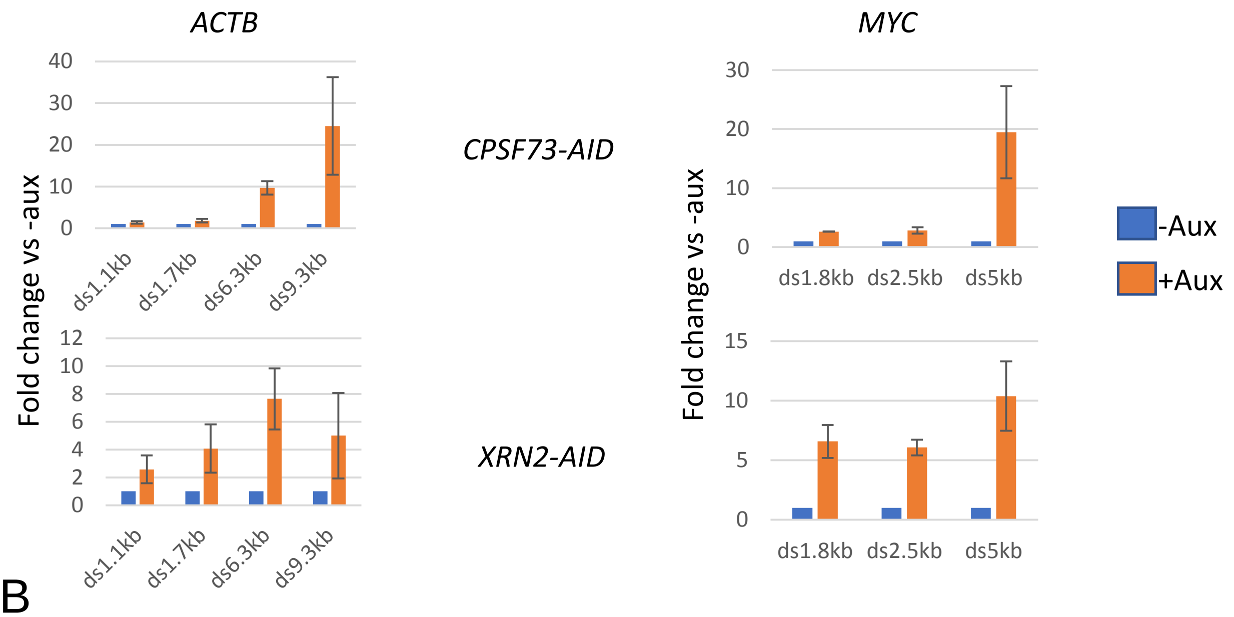
ACTB
MYC
CPSF73-AID
XRN2-AID
Fold change vs -aux
ds1.1kb
ds1.7kb
ds6.3kb
ds9.3kb
ds1.8kb
ds2.5kb
ds5kb
-Aux
+Aux

B

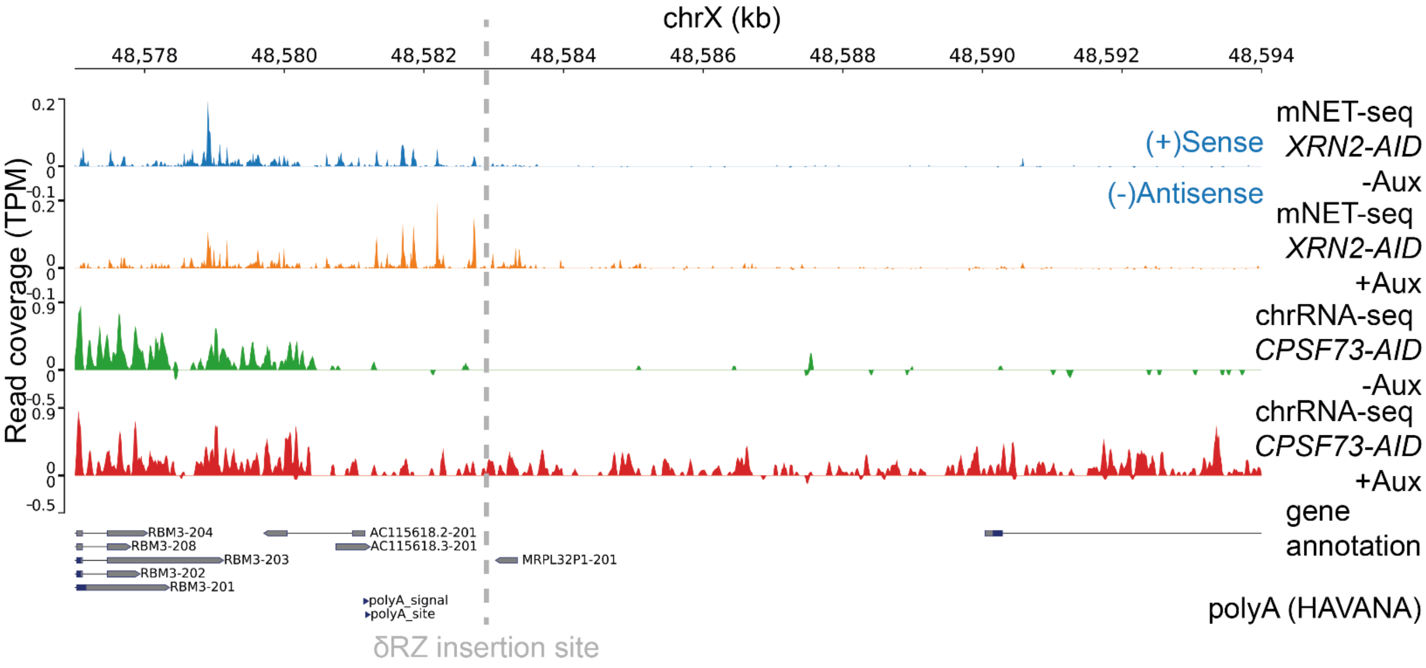
chrX (kb)
48,578
48,580
48,582
48,584
48,586
48,588
48,590
48,592
48,594
Read coverage (TPM)
mNET-seq
XRN2-AID
-Aux
(+)Sense
(-)Antisense
mNET-seq
XRN2-AID
+Aux
chrRNA-seq
CPSF73-AID
-Aux
chrRNA-seq
CPSF73-AID
+Aux
gene annotation
polyA (HAVANA)
RBM3-204
RBM3-208
RBM3-203
RBM3-202
RBM3-201
AC115618.2-201
AC115618.3-201
MRPL32P1-201
polyA_signal
polyA_site
δRZ insertion site

C

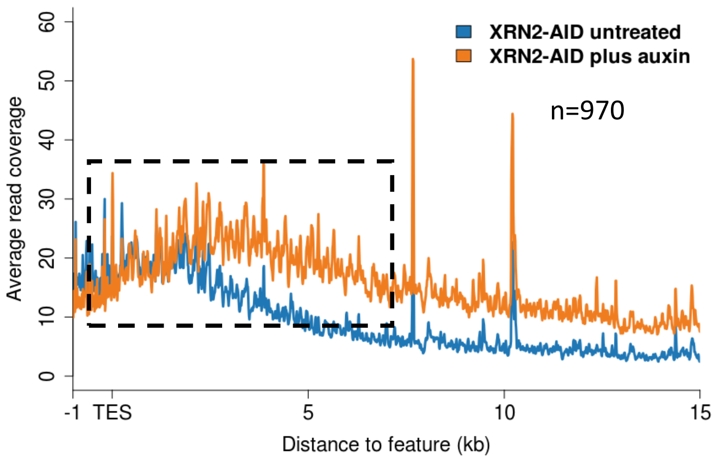
XRN2-AID untreated
XRN2-AID plus auxin
n=970
Average read coverage
-1
TES
5
10
15
Distance to feature (kb)

Supplemental Fig. S3: comparison of XRN2 and CPSF73 impacts at *MORF4L2* and *RBM3*

A: qRT-PCR analysis of 4-thiouridine labelled RNA from *ACTB* and *MYC* performed in *CPSF73-AID* or *XRN2-AID* cells treated or not with auxin (3h). Graph shows fold change of RNA level at each amplicon following normalisation to signal upstream of the PAS (ACTB US/MYC US) in the respective cell lines not treated with auxin. n=3. Error bars are SEM. Note that RNA was fragmented prior to streptavidin capture to prevent the purification of unlabelled parts of contiguous transcripts.

B: Chromosomal snapshot of *RBM3* derived from chromatin RNA-seq of *CPSF73-AID* cells treated or not with auxin (3h) and from mNET-seq of *XRN2-AID* cells treated or not with auxin (2h). Grey dotted line shows RZ insertion site.

C: Metagene analysis of our previously generated mNET-seq analysis of Pol II in *XRN2-AID* cells treated or not with auxin (2h) (Eaton et al., Genes and Development 2018). This plot shows signals normalised to gene body reads. The dashed box demarks a hump that is consistent with piled up Pol II being a general consequence of XRN2 loss.

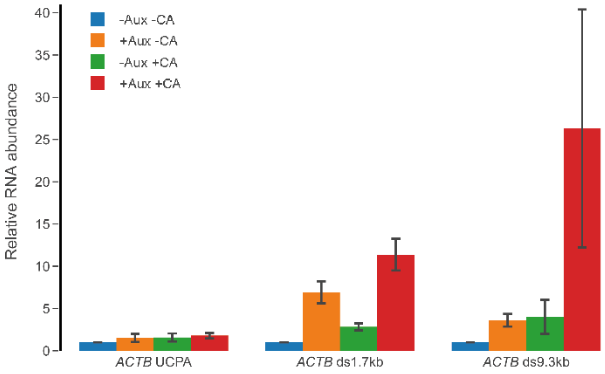

Supplemental Fig. S4: effects of calyculin A on *ACTB* transcriptional read-through

qRT-PCR analysis of *ACTB* read-through transcription in *XRN2-AID* cells that were either untreated or treated with auxin, calyculin A (CA) or both (all 1h). Graph shows RNA fold change relative to that found in untreated cells following normalisation to spliced ACTB. n=3. Error bars are SEM. Note that CA and auxin were added for just 1h because we noticed an effect of longer CA treatments on cell morphology. This short treatment explains the slight reduction in the effects of adding auxin alone versus the effects reported in figure 5, for tautomycetin, where auxin was added for 2h.

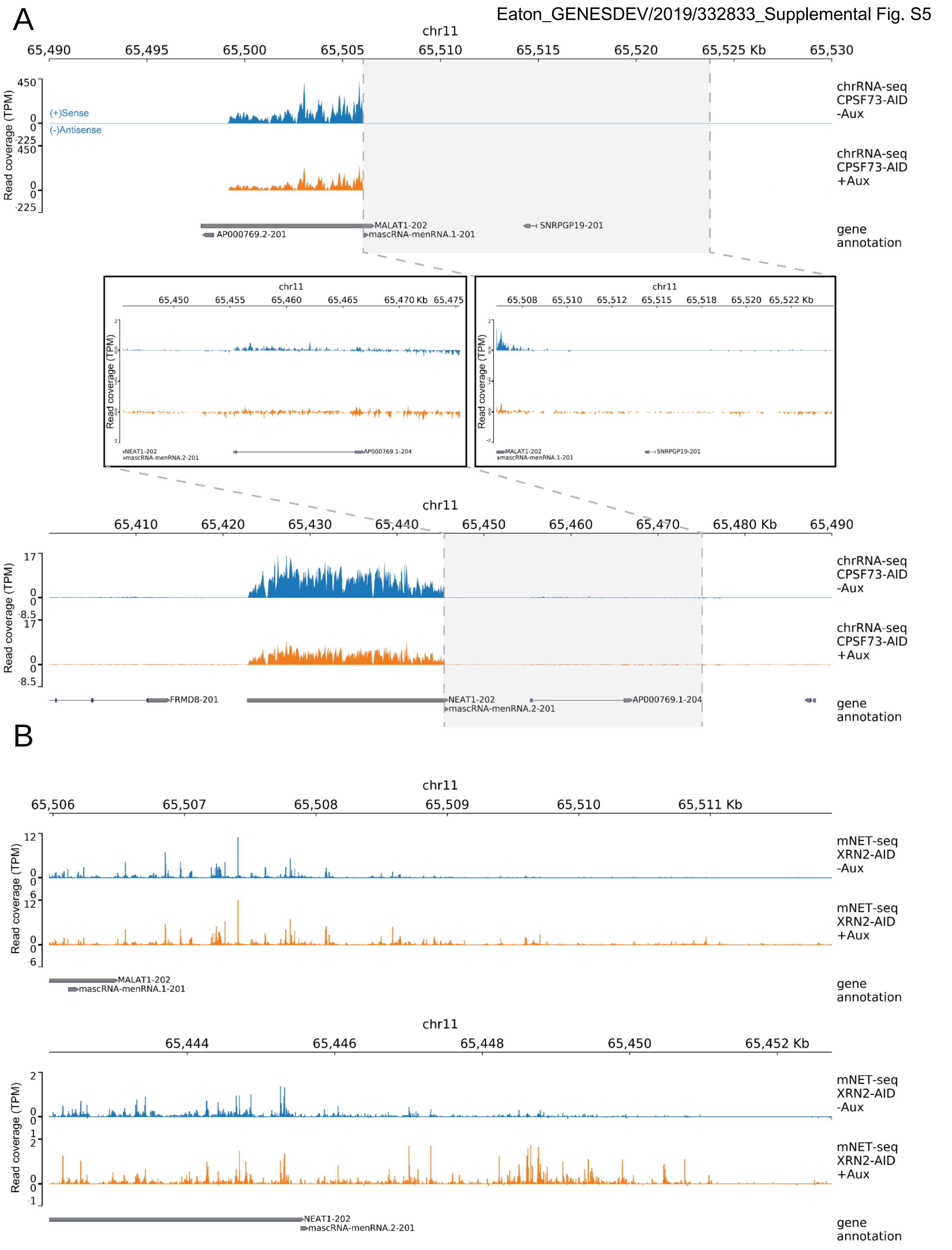
A
chr11
65,490
65,495
65,500
65,505
65,510
65,515
65,520
65,525 Kb
65,530
Read coverage (TPM)
(+)Sense
(-)Antisense
chrRNA-seq CPSF73-AID -Aux
chrRNA-seq CPSF73-AID +Aux
MALAT1-202
mascRNA-menRNA.1-201
AP000769.2-201
SNRPGP19-201
gene annotation
NEAT1-202
mascRNA-menRNA.2-201
AP000769.1-204
FRMD8-201
65,410
65,420
65,430
65,440
65,450
65,460
65,470
65,480 Kb
B
65,506
65,507
65,508
65,509
65,510
65,511 Kb
mNET-seq XRN2-AID -Aux
mNET-seq XRN2-AID +Aux
65,444
65,446
65,448
65,450
65,452 Kb

Supplemental Fig. S5: comparison of XRN2 and CPSF73 impacts at *MALAT1* and *NEAT1*

A: Chromosomal snapshot derived from chromatin RNA-seq of *CPSF73-AID* cells treated or not with auxin (3h) showing ncRNAs *NEAT1* and *MALAT1* whose 3' end can be cleaved by RNAseP. These transcripts do not show any read-through. A zoomed in snapshot of the 3' flank is shown (inset boxes).

B: mNET-seq traces of Pol II over *NEAT1* and *MALAT1* in *XRN2-AID* cells treated or not with auxin (2h). Both genes show a termination defect in the absence of XRN2.
